# Nanocages engineered from *Bacillus Calmette-Guerin* facilitate protective Vγ2Vδ2 T cell immunity against *Mycobacterium tuberculosis* infection

**DOI:** 10.1186/s12951-021-01234-3

**Published:** 2022-01-15

**Authors:** Jiang Pi, Zhiyi Zhang, Enzhuo Yang, Lingming Chen, Lingchan Zeng, Yiwei Chen, Richard Wang, Dan Huang, Shuhao Fan, Wensen Lin, Hongbo Shen, Jun-Fa Xu, Gucheng Zeng, Ling Shen

**Affiliations:** 1 Department of Clinical Immunology, Institute of Laboratory Medicine, School of Medical Technology, The First Dongguan Affiliated Hospital, Guangdong Provincial Key Laboratory of Medical Molecular Diagnostics, Guangdong Medical University, Dongguan, 523808 China; 2 Department of Microbiology and Immunology, University of Illinois at Chicago, Chicago, IL 60612 USA; 3 Department of Microbiology, Zhongshan School of Medicine, Key Laboratory for Tropical Diseases Control of the Ministry of Education, Sun Yat-Sen University, Guangzhou, 510080 Guangdong China; 4 Clinical Research Center, Department of Medical Records Management, Guanghua School of Stomatology, Hospital of Stomatology, Sun Yat-Sen University, Guangzhou, 510080 Guangdong China; 5 Clinic and Research Center of Tuberculosis, Shanghai Key Lab of Tuberculosis, Shanghai Pulmonary Hospital, Institute for Advanced Study, Tongji University School of Medicine, Shanghai, China

**Keywords:** Tuberculosis, *Bacillus Calmette-Guerin* (BCG), Nanocage, Vγ2Vδ2 T cells

## Abstract

Tuberculosis (TB), induced by *Mycobacterium tuberculosis* (Mtb) infection, remains a top killer among infectious diseases. While *Bacillus Calmette-Guerin* (BCG) is the sole TB vaccine, the clumped-clustered features of BCG in intradermal immunization appear to limit both the BCG protection efficacy and the BCG vaccination safety. We hypothesize that engineering of clumped-clustered BCG into nanoscale particles would improve safety and also facilitate the antigen-presenting-cell (APC)’s uptake and the following processing/presentation for better anti-TB protective immunity. Here, we engineered BCG protoplasts into nanoscale membraned BCG particles, termed as “BCG-Nanocage” to enhance the anti-TB vaccination efficiency and safety. BCG-Nanocage could readily be ingested/taken by APC macrophages selectively; BCG-Nanocage-ingested macrophages exhibited better viability and developed similar antimicrobial responses with BCG-infected macrophages. BCG-Nanocage, like live BCG bacilli, exhibited the robust capability to activate and expand innate-like T effector cell populations of Vγ2+ T, CD4+ T and CD8+ T cells of rhesus macaques in the ex vivo PBMC culture. BCG-Nanocage immunization of rhesus macaques elicited similar or stronger memory-like immune responses of Vγ2Vδ2 T cells, as well as Vγ2Vδ2 T and CD4+/CD8+ T effectors compared to live BCG vaccination. BCG-Nanocage- immunized macaques developed rapidly-sustained pulmonary responses of Vγ2Vδ2 T cells upon Mtb challenge. Furthermore, BCG- and BCG-Nanocage- immunized macaques, but not saline controls, exhibited undetectable Mtb infection loads or TB lesions in the Mtb-challenged lung lobe and hilar lymph node at endpoint after challenge. Thus, the current study well justifies a large pre-clinical investigation to assess BCG-Nanocage for safe and efficacious anti-TB vaccination, which is expected to further develop novel vaccines or adjuvants.

**Graphical Abstract:**

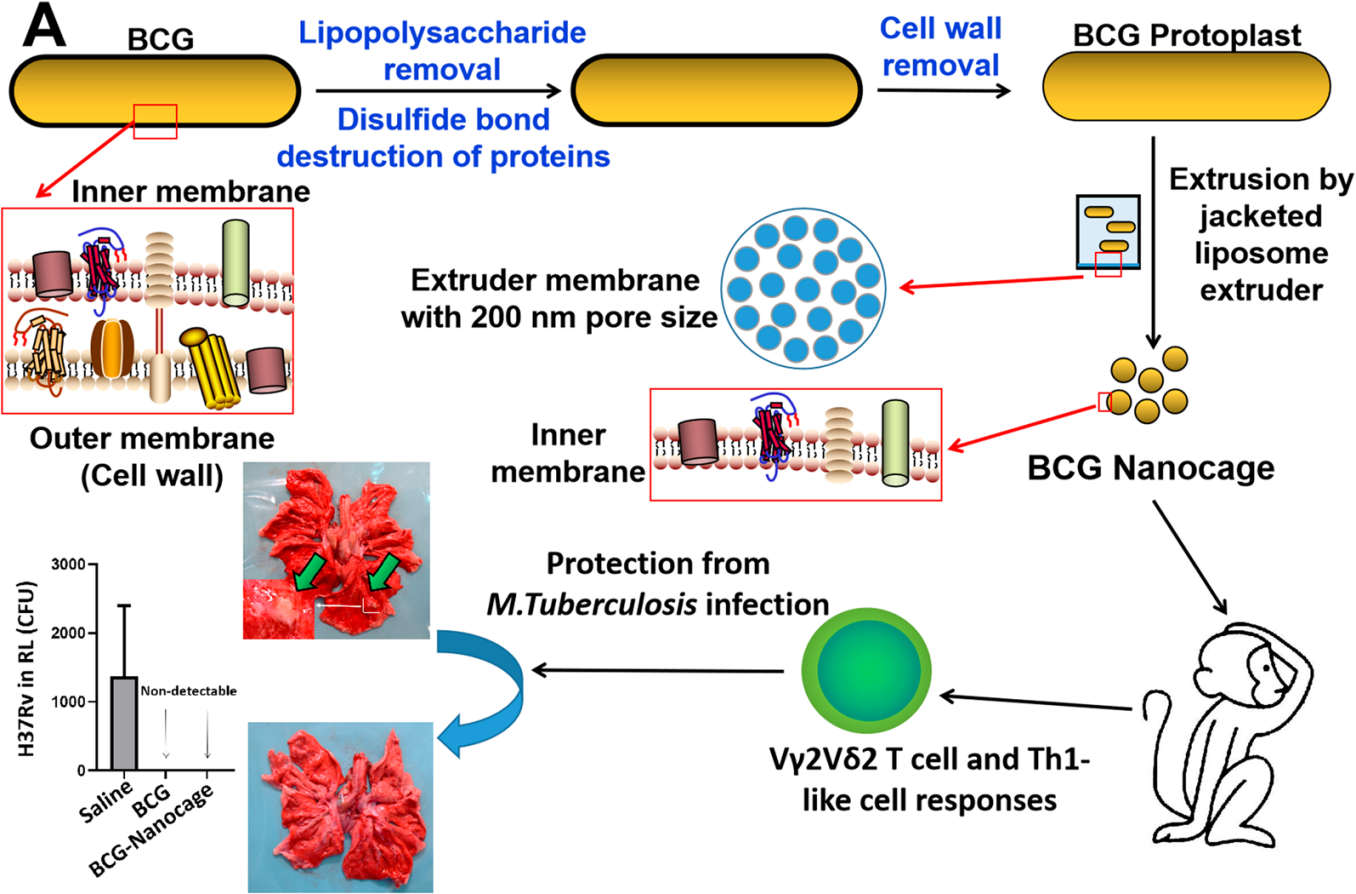

**Supplementary Information:**

The online version contains supplementary material available at 10.1186/s12951-021-01234-3.

## Background

Tuberculosis (TB), induced by *Mycobacterium tuberculosis* (Mtb) infection, remains one of the top killers among infectious diseases world widely with 1.5 million deaths in 2020 as reported by the global tuberculosis report 2021. Despite global efforts to halt the spread of TB, the lack of an effective vaccine, limited cure rates for drug-resistant Mtb (MDR-TB), and HIV/AIDS co-infection make TB remain a leading global public health threat [1]. Therefore, the development of novel vaccines and therapeutics is crucial to overcome the global burdens of TB. *Bacille Calmette-Guerin* (BCG), a live attenuated form of *Mycobacterium bovis,* is widely used as the only vaccine against TB in newborn babies in most of countries [2]. BCG vaccination has been proved to be, although variable, but still highly effective in preventing TB meningitis and disseminated TB in children [3, 4]; however, the efficacy of BCG vaccinations in newborns appears to be waning or disappearing in young adults, and BCG vaccinations in adults are hard to induce similar protections as like in newborns [5]. Although there are several hypotheses to explain the limitation or failure of BCG vaccinations to mount more effective protection against pulmonary TB [6], the exact mechanisms underlying this insufficient protection efficacy of BCG vaccination remain largely unclear.

Due to the controversial protective efficacy of BCG vaccination against TB in adults, thousands of researchers have spent their life time in developing more effective TB vaccines. Up to 2020, there are already 16 TB vaccine candidates at different phases (from phase 1 to phase 3) in clinical trials, including viable whole-cell vaccines, inactivated whole-cell vaccines and adjuvanted protein subunit vaccine [7, 8]. Unfortunately, there are still no TB vaccine candidates are approved for TB protection up to now, except for the widely used BCG. However, the efficacy and safety of BCG vaccine still remain critical concerns, especially for those individuals with immune-compromised conditions such as HIV/AIDS [9, 10]. Regional lymphadenitis and suppurative lymphadenitis have been reported as the most frequent adverse effects for live BCG vaccination in infants, which may lead to the hospitalization of infants [11, 12]. Thus, increasing attentions have been paid to explore new ways to enhance the efficacy, reduce the side effects and safety risk of BCG vaccine in recent years. Although a recent study demonstrated that intravenous vaccination of nonhuman primates was much more immunogenic and protective against TB than the licensed intradermal immunization route [13], the unapproved intravenous administration of live BCG would raise a greater concern about BCG safety due to the wide-spread BCG dissemination introduced by intravenous injection. Additionally, it’s very difficult to control the level of vaccine-induced immunological responses in peripheral blood, where some immune cell subsets, such as T cells and NK cells, can directly react with the potent immunogens in vaccine. Thus, intravenous injection is still rarely used for vaccine administration, while subcutaneous injection and intramuscular injection are thought to be more safety strategy for vaccine administration. Based on these facts, how to engineer BCG into a kind of more effective and safe vaccine that may suitable for subcutaneous or intramuscular vaccination to activate more potent immunological responses with less side effects and lower safety risk remains a big challenge.

Both Mtb and *Mycobacterium bovis-*derived BCG are the most successful evolutionary bacteria in Earth, which exhibits clumped-clustered features due to their extremely thick, waxy, and complex cellular wall. While the significance of such clumped-clustered features for evolutionary advantage of Mtb or BCG are not exactly clear, but, physically, these clumped-clustered features will significantly increase the size (increase from microscale single bacterium to millimeter scale clustered or brunched bacteria) and steric barrier of Mtb or BCG against host cells. Such features may therefore contribute to their escape from the surveillance or engulfment machinery and following bacterial digestion of host cells. However, as shown by transmission electron microscopy (TEM) imaging of Mtb-infected macrophages, most of innate immune cellular machinery in host cells for antigen processing and presentation of macrophages, such as phagosomes and lysosomes, are at nanoscale [14–16]. Thus, these clumped-clustered features of Mtb and BCG may severe as physical or size barriers leading to impaired efficacy and efficiency of macrophages for bridging the innate and adaptive immune programing involving in bacterial recognition/lysis into antigens and presentation of antigens to initiate an effective anti-TB T cell immunity. Additionally, these clumped-clustered features might also lead to some safety issues of BCG for vaccination.

Such a postulation of size-effect- or steric-effect- driven mechanism of Mtb/BCG to escape innate/adaptive immune surveillance can also be supported by classical bacterial biofilm theory, by which bacteria may utilize larger-size (usually micro- or millimeter scale) biofilm to better survive from immune surveillance and harsh environment [17, 18]. Furthermore, biomaterials at nanoscale might be more efficiently involved in antigen processing and presentation machinery [19], as most of these sub-cellular machineries are at nanoscale. In recent decades, engineering nanoparticles prepared based on the nanobiotechnology methodology have attracted plenty of attentions worldwide not only in cancer, but also in infectious diseases [20, 21]. We have also recently shown that nanoparticles could promote the phagosome-lysosome fusion or improve delivery of anti-TB drugs into phagosome of Mtb-infected macrophages, and therefore significantly enhance efficacy of anti-TB drugs [22, 23]. Bacteria-derived nanovesicles and virus-like nanoparticles have also been developed into promising nanovaccines or drug nanocarriers [24–26], indicating the potentials of nanobiotechnology to engineer BCG. Thus, we hypothesize that engineering of milli/ micrometer scale, brunched, clustered BCG into nanoscale particles would facilitate the immunological processes from bacterial recognition and digestion to antigen presentation by macrophages and therefore facilitate the innate and adaptive immune programming to induce more potent or broader protective T cell immune responses.

In this work, combining our decade-long TB immunology [27–31] and nanotechnology [16, 22, 32–34] expertise, we use live BCG bacilli to develop a bioinspired nanoparticle, termed as BCG-Nanocage. Compared with live BCG, this BCG-Nanocage engineered from live BCG show outstanding macrophage-targeting effects, reduced cytotoxicity after treatment with macrophages and, more importantly, BCG-Nanocage not only maintain similar immune responses of CD4+ /CD8+ T subsets but also induce stronger Vγ2Vδ2 T cell immune responses that are associated with sterilized Mtb burdens and much milder pulmonary TB pathology in Mtb-infected rhesus macaques. Thus, this work may open a new avenue for development of novel TB vaccines and therapeutics.

## Methods

### Preparation and characterization of BCG-Nanocage

*Bacillus Calmette-Guerin* (BCG, ATCC, Catalog No. 35734) at logarithmic phase were treated with 0.1 M EDTA (Sigma, Catalog No. 03690) and 0.1% β-mercaptoethanol (Sigma, Catalog No. 444203) at 37 ℃ for 2 h shaking. After washed, the obtained BCG were then treated with 20 mg/ml lysozyme (Sigma, Catalog No. L1667) in phosphate buffer saline (PBS) solution for overnight shaking at 37 ℃ to obtain BCG protoplasts. After suspended in PBS, the BCG protoplasts were dispersed and then extruded sequentially through the 1000 nm and 200 nm sized polycarbonate membrane filters by a nitrogen gas-driven Jacketed Liposome Extruder (Genizer). The obtained products were centrifuged at 20,000*g* for 30 min to exclude the large fragments. The BCG-Nanocage were collected by ultracentrifugation at 100,000*g* for 2 h at 4 ℃, washed with Tris-buffered saline (pH 7.2), pelleted down by 100,000*g* for 2 h at 4 ℃. After that, BCG-Nanocage engineered from BCG (ATCC, Catalog No. 35734) was aliquoted and stored at −80 ℃ until experimental use. For green fluorescent protein (GFP) tagged BCG-Nanocage (GFP-BCG-Nanocage) preparation, similar method was used except that GFP-BCG (Green fluorescence protein tagged BCG bacteria) were used instead of BCG. The obtained BCG-Nanocage were analyzed by dynamic light scattering (DLS), and characterized by transmission electron microscopy (TEM) after methyl cellulose-uranyl acetate staining and scanning electron microscopy (SEM) after gold coating. For protein contents analysis, BCG and BCG-Nanocage were lysed by ultrasound treatment, and then subjected for SDS-PAGE analysis by Coomassie Blue staining.

### Viability of THP-1 macrophages

1 × 10^4^ THP-1 cells were seeded into 96-well plates for 48 h incubation with 100 nM PMA stimulation in incubator at 37 ℃ with 5% CO_2_. Then, BCG and BCG-Nanocage with the same protein contents to BCG were added for 3 days of incubation in an incubator at 37 ℃ with 5% CO_2_. After incubated with MTT for 4 h, the supernatant was discarded, followed by the addition of 150 μl DMSO and absorbance measurements by a microplate reader at 570 nm.

### Effects of BCG-Nanocage on macrophage polarization and intracellular cytokines production in THP-1 macrophages

1 × 10^6^ THP-1 cells were seeded into 6 well plates for 48 h incubation with 100 nM PMA stimulation in incubator at 37 ℃ with 5% CO_2_. Then, BCG and BCG-Nanocage with the same protein contents to BCG were added for 3 days of incubation in incubator at 37 ℃ with 5% CO_2_. For macrophage polarization analysis, the collected cells were washed and then stained with anti-human CD11b-APC antibody (Biolegend, Catalog No. 101211), anti-human CD14-Percp-Cyc5.5 antibody (BD, Catalog No. 561116), anti-human CD80-Alexa Fluor® 488 antibody (Biolegend, Catalog No. 305214) and anti-human CD206-Alexa Fluor® 700 antibody (Biolegend, Catalog No. 321132) for 30 min at room temperature in dark. The washed cells were then permeabilized for 45 min with Cytofix/cytoperm (BD, Catalog No. 554714) and stained for another 30 min with anti-human IFN-γ-EF450 antibody (ThermoFisher, Catalog No. 48-7319-42), anti-human TNF-α-PE antibody (Biolegend, Catalog No. 502909) and anti-human CAP-18 Alexa Fluor® 594 antibody (Santa Cruz Bio, Catalog No. sc-130552 AF594) at 4 ℃, followed by re-suspending in 4% formaldehyde-PBS for flow cytometry analysis (BD, LSRFortessa).

### Isolation of peripheral blood mononuclear cell (PBMC) and bronchoalveolar lavage (BAL) lymphocytes from rhesus macaques

Following sedation of macaques with ketamine and xylazine, bronchoalveolar lavage (BAL) and fluid collection were carried out using a pediatric bronchoscope as previously described [27, 28], The bronchoscope was inserted into the bronchial branches distributing to the infected right caudal and other lung lobes of the animals to allow for harvesting of cells, including lymphocytes, in the airway. Isolation of lymphocytes from BAL fluid and peripheral blood mononuclear cell (PBMC) from EDTA blood was done as previously described [35].

### Cytotoxicity of BCG-Nanocage on T cells, B cells, NK cells and macrophages

Fresh PBMC from rhesus monkey were seeded into 96 well plates with a density of 1 × 10^6^ cells/well. Then, 4 × 10^5^ CFU of BCG and 1 μg/ml of BCG-Nanocage (Protein concentration) were added for 6 days incubation in an incubator at 37 ℃ with 5% CO_2_. The collected cells were then stained with anti-human CD3-PerCP antibody (BD, Catalog No. 552851), anti-human CD14-AF700 antibody (Biolegend, Catalog No. 301822), anti-human CD80-PE antibody (Biolegend, Catalog No. 305208), anti-human CD16-BV510 antibody (Biolegend, Catalog No. 302048), anti-human CD20-BV421 antibody (BD, Catalog No. 562873), anti-human CD56-PE-Cy7 antibody (Biolegend, Catalog No. 318318), anti-human Vγ2-FITC antibody (Thermo Fisher, Catalog No. TCR2720), anti-human CD161-APC antibody (Biolegend, Catalog No. 339912), anti-human CD4-BV605 antibody (Biolegend, Catalog No. 317438), anti-human CD8-APC-H7 antibody (BD, Catalog No. 560179) at room temperature for 30 min. After wash, cells were permeabilized for 45 min with Cytofix/cytoperm (BD, Catalog No. 554714) and stained for another 45 min with anti-human IFN-γ-PECF-594 antibody (BD, Catalog No. 562392), followed by re-suspending in 4% formaldehyde-PBS for flow cytometry analysis.

### Ex vivo co-stimulation of PBMC by BCG-Nanocage/IL2

BCG/IL2 or BCG-Nanocage/IL2 co-stimulation were applied in PBMC to determine the activation and expansion of T cells. Briefly, 1 × 10^6^ PBMC isolated from rhesus macaques were cultured in U-bottomed 96-well plates in the absence or presence of 1 × 10^5^ colony forming unit (CFU) BCG or BCG-Nanocage with the same protein contents to BCG, and then supplemented at day 0 and 3 with 20 U/mL hIL-2 (Sigma-Aldrich, Catalog No. I17002). At day 6, cells were treated with brefeldin A for 4 h, and then stained with anti-human CD3-PE-Cy™7 antibody (BD, Catalog No. 563423), anti-human CD8-APC-H7 antibody (BD, Catalog No. 560179), anti-human Vγ2-FITC antibody (Thermo Fisher, Catalog No. TCR2720), anti-human CD14-AF700 antibody (Biolegend, Catalog No. 301822), anti-human CD161-BV510 antibody (Biolegend, Catalog No. 339922), anti-human CD4-BV711 antibody (Biolegend, Catalog No. 317440), anti-human TNF-a-BV605 antibody (Biolegend, Catalog No. 502936), anti-human NKG2C lgG antibody (Miltenyi Biotec, Catalog No. 130-120-588) at room temperature for 30 min, followed by anti-biotin PerCP/Cyanine5.5 Streptavidin (Biolegend, Catalog No. 405214) incubation for 20 min. After wash, cells were permeabilized for 45 min with Cytofix/cytoperm (BD, Catalog No. 554714) and stained for another 45 min with anti-human Perforin-PF647P antibody (MABTECH, Catalog No. 3465-72-100T), anti-human Granzyme B-PB antibody (BioLegend, Catalog No. 515408), anti-human Granulysin-PE antibody (eBioscience, Catalog No. 12-8828-42) and anti-human IFN-γ-PECF-594 antibody (BD, Catalog No. 562392), followed by re-suspending in 4% formaldehyde-PBS for flow cytometry analysis.

### Cellular uptake of GFP-BCG-Nanocage by T cells and macrophages in Bronchoalveolar lavage (BAL) of macaques

Bronchoalveolar lavage (BAL) isolated from rhesus macaques were seeded into 96 well plates with a density of 8 × 10^5^ cells/well. Then, GFP-BCG and GFP-BCG-Nanocage with the same protein contents to GFP-BCG were added for 12 h incubation in an incubator at 37 ℃ with 5% CO_2_. The collected cells were washed and then pass through 70 μm filter, followed by the staining with anti-human CD3-PE antibody (BD, Catalog No. 552127) and anti-human CD11b-APC antibody (Biolegend, Catalog No. 101212) for 10 min at room temperature in dark. After washed, cells were dropped onto the confocal dish for 20 min, and then used for confocal microscopy (Zeiss, LSM710) imaging immediately.

### Cellular uptake of GFP-BCG-Nanocage by T cells, B cells, endothelium and macrophages in intraepithelial lymphocytes (IEL) of macaques

Intraepithelial lymphocyte (IEL) from the small intestine of H37Rv infected macaques was seeded into 96 well plates with a density of 1 × 10^6^ cells/well. Then, GFP-BCG and GFP-BCG-Nanocage with the same protein contents to GFP-BCG were added for 1 h or 3 h incubation in an incubator at 37 ℃ with 5% CO_2_. The collected cells were washed and then stained with anti-human anti-human CD3-PE antibody (BD, Catalog No. 552127), anti-human CD20-APC antibody (Biolegend, Catalog No. 302310) and anti-human CD14-Percp-Cyc5.5 antibody (BD, Catalog No. 561116) at room temperature for 30 min. Cells were analyzed by flow cytometry (BD, LSRFortessa) after fixation with 4% formaldehyde-PBS.

### Macaque and institutional animal care and use committee approval

Female and male rhesus macaques aged 4–8 year were used in the current study. All macaques had negative routine PPD TB test results. The use of macaques and all experimental procedures were approved by Institutional Animal Care and Use Committee and Biosafety Committees at University of Illinois at Chicago.

### Immunization of macaques with BCG and BCG-Nanocage

A total of 2.5 × 10^5^ CFU of BCG in 0.1 ml saline were intracutaneously injected for immunization following the typical BCG immunization strategy at the left arm of two macaques. 0.1 ml of BCG-Nanocage with same protein concentration with 2.5 × 10^5^ CFU of BCG were subcutaneously injected at the left arm of another two macaques as prime immunization, and further subcutaneously injected at the left arm of these macaques as boost immunization after 4 weeks of prime immunization.

### Staining of Vδ2Vγ2+ T cells in the lymphocytes from macaques

Freshly isolated lymphocytes were stained with anti-human Vδ2 antibody (Thermo Fisher, Catalog No. TCR1732) at room temperature for 20 min. After wash, cells were then stained with PB-Goat anti-human secondary antibody (Invitrogen, Catalog No.) at room temperature for 15 min. After incubated with mouse serum (Santa Cruz Biotechnology, Catalog No. sc-45051) at room temperature for 20 min, cells were further stained with anti-human CD3-PE-Cy™7 antibody (BD, Catalog No. 557749) and anti-human Vγ2-FITC antibody (Thermo, Catalog No. TCR2720) at room temperature for 20 min. After wash, cells were re-suspended in 4% formaldehyde-PBS for flow cytometry analysis.

### Detection of intracellular cytokines and cytotoxic molecules in T cells with ex vivo co-stimulation with anti-CD28/anti-CD49d + BCG-Nanocage

Cell surface marker staining and intracellular cytokine staining (ICS) was done in PBMC of rhesus macaques before and after BCG immunization or BCG-Nanocage boost immunization. A total of 1 × 10^6^ PBMC plus 1 mg/ml of anti-human CD28 antibody (BD, Catalog No. 556620) and 1 mg/ml of anti-human CD49d antibody (BD, Catalog No. 555502) was incubated with BCG-Nanocage (1 μg/ml), or media alone in 200 ml final volume for 1 h at 37 ℃, 5% CO_2_, followed by an additional 4 h incubation in the presence of brefeldin A (BD, Catalog No. BDB555029). The collected cells were then stained with anti-human CD3-PE-Cy™7 antibody (BD, Catalog No. 563423), anti-human CD8-APC-H7 antibody (BD, Catalog No. 560179), anti-human CD4-BV711 antibody (Biolegend, Catalog No. 317440), anti-human Vγ2-FITC antibody (Thermo Fisher, Catalog No. TCR2720), anti-human CD86-BV510 antibody (Biolegend, Catalog No. 305432), anti-human CD14-AF700 antibody (Biolegend, Catalog No. 301822), anti-human CD80-PerCP-Cy5.5 antibody (Biolegend, Catalog No. 305232) and anti-human CD161-BV605 antibody (Biolegend, Catalog No. 339916) for 30 min. After wash, cells were permeabilized for 45 min with Cytofix/cytoperm (BD, Catalog No. 554714) and stained for another 45 min with anti-human Perforin-PF647P antibody (MABTECH, Catalog No. 3465-72-100T), anti-human Granzyme B-PB antibody(BioLegend, Catalog No. 515408), anti-human Granulysin-PE antibody(eBioscience, Catalog No. 12-8828-42) and anti-human IFN-γ-PECF-594 antibody (BD, Catalog No. 562392), followed by re-suspending in 4% formaldehyde-PBS for flow cytometry analysis.

### Mtb infection of rhesus macaques

After 4 weeks of boost BCG-Nanocage immunization, two macaques in each group were sedated with ketamine and xylazine by i.m. injection. A pediatric bronchoscope was inserted into the right caudal lung lobe of the animals, and 5 CFU of Mtb H37Rv strain was injected in 3 mL of saline followed by a 3-mL bolus of air to ensure full dose administration. The colony-forming unit dose for infection was confirmed by careful post-inoculation titration on a Middle brook 7H11 plate (Becton Dickinson, Catalog No. 297250) as previously described [31].

### Direct intracellular cytokine staining (ICS) analysis of PBMCs and BAL Lymphocytes

Freshly isolated lymphocytes from macaques were stained with anti-human CD3-PE-Cy™7 antibody (BD, Catalog No. 563423), anti-human CD8-APC-H7 antibody (BD, Catalog No. 560179), anti-human Vγ2-FITC antibody (Thermo Fisher, Catalog No. TCR2720), anti-human CD14-AF700 antibody (Biolegend, Catalog No. 301822), anti-human CD80-PE antibody (Biolegend, Catalog No. 305208), anti-human CD161-APC antibody (Biolegend, Catalog No. 339912), anti-human CD4-BV605 antibody (Biolegend, Catalog No. 317438), anti-human CD11b-APC antibody (Biolegend, Catalog No. 101212), anti-human CD86-BV510 antibody (Biolegend, Catalog No. 305432), anti-human HLA-DR-PerCP-Cy5.5 antibody (Biolegend, Catalog No. 307630) and anti-human CD69-BV421 antibody (Biolegend, Catalog No. 310930) at room temperature for 30 min. After wash, cells were permeabilized for 45 min with Cytofix/cytoperm (BD, Catalog No. 554714) and stained for another 45 min with anti-human IFN-γ-PECF-594 antibody (BD, Catalog No. 562392), followed by re-suspending in 4% formaldehyde-PBS for flow cytometry analysis.

### Determination of bacterial loads in tissues

Lung tissues of macaques were harvested and processed for Mtb colony-forming unit determination as described previously [27, 31]. Briefly, tissue homogenates were made using a homogenizer (PRO 200; PRO Scientific) and were diluted using sterile PBS + 0.05% Tween-80. Fivefold serial dilutions of samples were plated on Middle brook 7H11 plates (Becton Dickinson, Catalog No. 297250). The colony-forming unit counts on plates were measured after 3–4 weeks of culture.

### Gross pathologic and histologic analysis of Mtb-infected macaques at necropsy

Multiple tissue specimens were collected from all organs whether or not they showed gross lesions. For organs with visible lesions, their number, location, size, distribution, and consistency were recorded. Microscopic analysis of TB lesions was applied similarly as we previously described [31]. Briefly, the extent of involvement for each lung lobe was determined using digital scans to record total pixel counts on H&E-stained material and specimen area measured in square cm using Image-Pro Plus software.

### Statistical analysis

Statistical analysis was done using one-way parametric test as indicate, p < 0.05 was considered as significant. All statistical analyses were conducted using GraphPad software.

## Results

### Development and characterization of nanoparticle-like BCG-Nanocage from microscale *Bacillus Calmette-Guerin* (BCG)

Presumably Nano-sized biomaterials are more readily ingested by antigen-presenting cells (APC) (e.g. Dentric cell (DC) and macrophages (MΦ)) than micro-sized biomaterials or clumped aggregates, and thus more effectively activate/differentiate T cells for robust effector functions and protection [36, 37]. In recent years, nanovesicles derived from bacterial protoplast open up a new avenue as an effective, safe delivery system to optimize current chemotherapy and immunization strategies both in cancers and infectious diseases [24, 25]. Thus we hypothesized that engineering of clumped-clustered BCG into nanoscale particles would improve safety even by intravenous administration and facilitate the antigen-presenting-cell (APC)’s uptake and processing/presentation of vaccine for better immunity against TB.

**Fig. 1:**
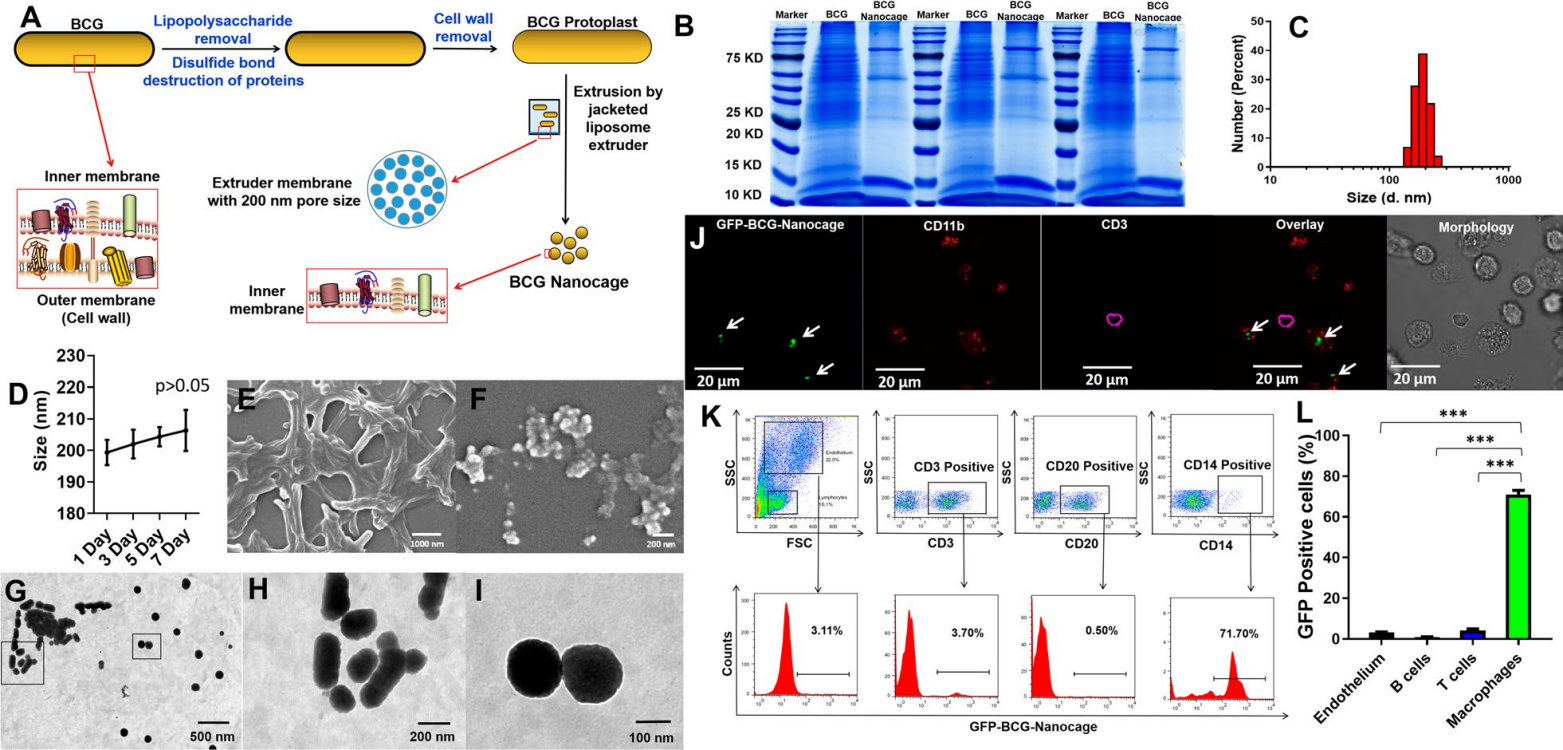
Development and characterization of BCG-Nanocage, and BCG-Nanocage preferentially target macrophages (MΦ), but not T cells, B cells or endothelium. **A** Schematic diagram of preparation for BCG-Nanocage from BCG. **B** SDS-PAGE analysis of the protein contents in BCG lysis and BCG-Nanocage lysis from three different batches of samples, proteins were stained by Coomassie Blue. **C** Size distribution of BCG-Nanocage. **D** Size distribution changes of BCG-Nanocage during 7 days storage at 4 ℃. **E** Scanning electron microscopy (SEM) imaging of BCG bacteria. **F** SEM imaging of BCG-Nanocage. **G** Transmission electron microscopy (TEM) imaging of BCG-Nanocage. **H**–**I** Enlarged TEM imaging of BCG-Nanocage from the indicated area in (**G**). **J** Confocal imaging for cellular uptake of GFP-BCG-Nanocage by macrophages from the freshly isolated bronchoalveolar lavage (BAL) fluid of rhesus macaques after 1 h treatment of GFP-BCG-Nanocage, followed with staining using anti-human CD11b-APC antibody and anti-human CD3-PE antibodies, scale bar: 20 μm. **K** Flow cytometry analysis for cellular uptake of GFP-BCG-Nanocage by macrophages, T cells, B cells and endothelium from the freshly isolated intraepithelium (IEL) of rhesus macaques after 1 h treatment. **L** Cellular uptake of GFP-BCG-Nanocage by macrophages, T cells, B cells and endothelium from the freshly isolated intraepithelial lymphocytes of rhesus macaques after 1 h treatment, n = 4, ***p < 0.001

To generate BCG-Nanocage, the outer membrane of BCG was deleted by treatment with EDTA and β-mercaptoethanol for lipopolysaccharide removal and disulfide bond destruction of membrane proteins, which would improve the sensitivity of BCG against lysozyme lysis (Fig. 1A). After that, the EDTA and β-mercaptoethanol treated BCG was treated with lysozyme to further catalyze the destruction of BCG cell wall by cleaving the peptidoglycan components and removing periplasmic components of cell wall (Fig. 1A). The obtained BCG protoplasts were then serially extruded through polycarbonate membranes with nano-sized pores to generate cage-like structures with BCG components inside at nanoscale (Fig. 1A). The obtained BCG-Nanocage with BCG intracellular components inside did not contain any live BCG, and no bacterial colonies were observed in BCG-Nanocage inoculated 7H11 plates (data not shown).

Firstly, to explore whether the main components of BCG were still maintained in BCG-Nanocage, we also analyzed the differences of protein components between BCG lysis and BCG-Nanocage lysis by SDS-PAGE gel. As shown in Fig. 1B, it was clearly that there were some missing proteins in BCG-Nanocage lysis compared with BCG lysis, which might be due to the removal of membrane contents during the preparation of BCG-Nanocage. And interestingly, we also found that some proteins were enriched in BCG-Nanocage (Fig. 1B), which might be some unknown BCG intracellular proteins. These results clearly implied that BCG-Nanocage still maintained most of the protein of BCG, even with some enriched proteins, which might be responsible for the anti-TB effects of BCG-Nanocage.

Dynamic light scattering (DLS) analyses showed that the average diameter of BCG-Nanocage was approximately 200 nm (Fig. 1C), with few size changes during 7 day storage at 4 ℃ (Fig. 1D). Long-time storage of BCG-Nanocage at −80 ℃ would resulted in partial increase of the particles size (Additional file 1: Fig. S1), which might be due to the influence of freezing and thawing processes. These manufacture procedures steadily made the micro-sized rod-shape BCG with branch- or clumped-clustered features (Fig. 1E) into dispersible nano-sized, nanoparticle-like BCG-Nanocage (Fig. 1F) as illustrated by SEM. TEM imaging also indicated uniform nanoparticle-like morphology of BCG-Nanocage (Fig. 1G–I). These results collectively indicated the successful engineering of branch- or cluster-like, microscale live BCG into nano-sized, nanoparticle-like BCG-Nanocage.

### BCG-Nanocage could readily be ingested/taken by antigen presenting cell macrophages (MΦ), but not T cells

Since BCG-Nanocage show features of uniform nano-sized particles upon removal of large parts of membrane materials of live BCG, we then asked whether such micro-to-nano scale shifting procedures would confer any change on their macrophage-targeting property. To determine the macrophage-targeting effects of BCG-Nanocage, we isolated bronchoalveolar lavage (BAL) from rhesus macaques and incubated with GFP-BCG-Nanocage and live GFP-BCG, respectively, to comparatively analyze the macrophage-targeting property between GFP-BCG-Nanocage and live GFP-BCG. As shown by confocal microscopic imaging (Fig. 1J), GFP-BCG-Nanocage, similar with GFP-BCG (Additional file 1: Fig. S2A), preferentially accumulated into CD11b+ macrophages, but not CD3+ T cells.

To further quantitatively demonstrate the macrophage-targeting effects of BCG-Nanocage, intraepithelium (IEL) were further isolated from rhesus macaques and incubated with GFP-BCG-Nanocage and live GFP-BCG, respectively, and then subjected for flow cytometry-based quantitative analysis of intracellular GFP contents. Quantitative flow cytometric analysis showed that, similar as GFP-BCG with the same protein contents (Additional file 1: Fig. S2B–C), GFP-BCG-Nanocage demonstrated much higher intracellular GFP signals in CD14+ macrophages) than those of CD20+ B cells, CD3+ T cells and endothelium (Fig. 1K–L). By comparing the intracellular GFP signals, we found that there were no significant differences in the cellular uptake of GFP-BCG and GFP-BCG-Nanocage in CD14+ macrophages, CD20+ B cells, CD3+ T cells and endothelium after 1 h treatment (Additional file 1: Fig. S3). However, the cellular uptake of GFP-BCG-Nanocage was much higher than that of GFP-BCG in macrophages after 3 h treatment, while no significant differences in the cellular uptake of GFP-BCG in CD20+ B cells, CD3+ T cells and endothelium (Additional file 1: Fig. S3), suggesting the more potent macrophage uptake of BCG-Nanocage after 3 h treatment. These qualitative and quantitative results further suggested the excellent macrophage-targeting properties of BCG-Nanocage.

### BCG-Nanocage-ingested macrophages (MΦ) exhibited better viability and greater antimicrobial responses than BCG-infected MΦ

**Fig. 2:**
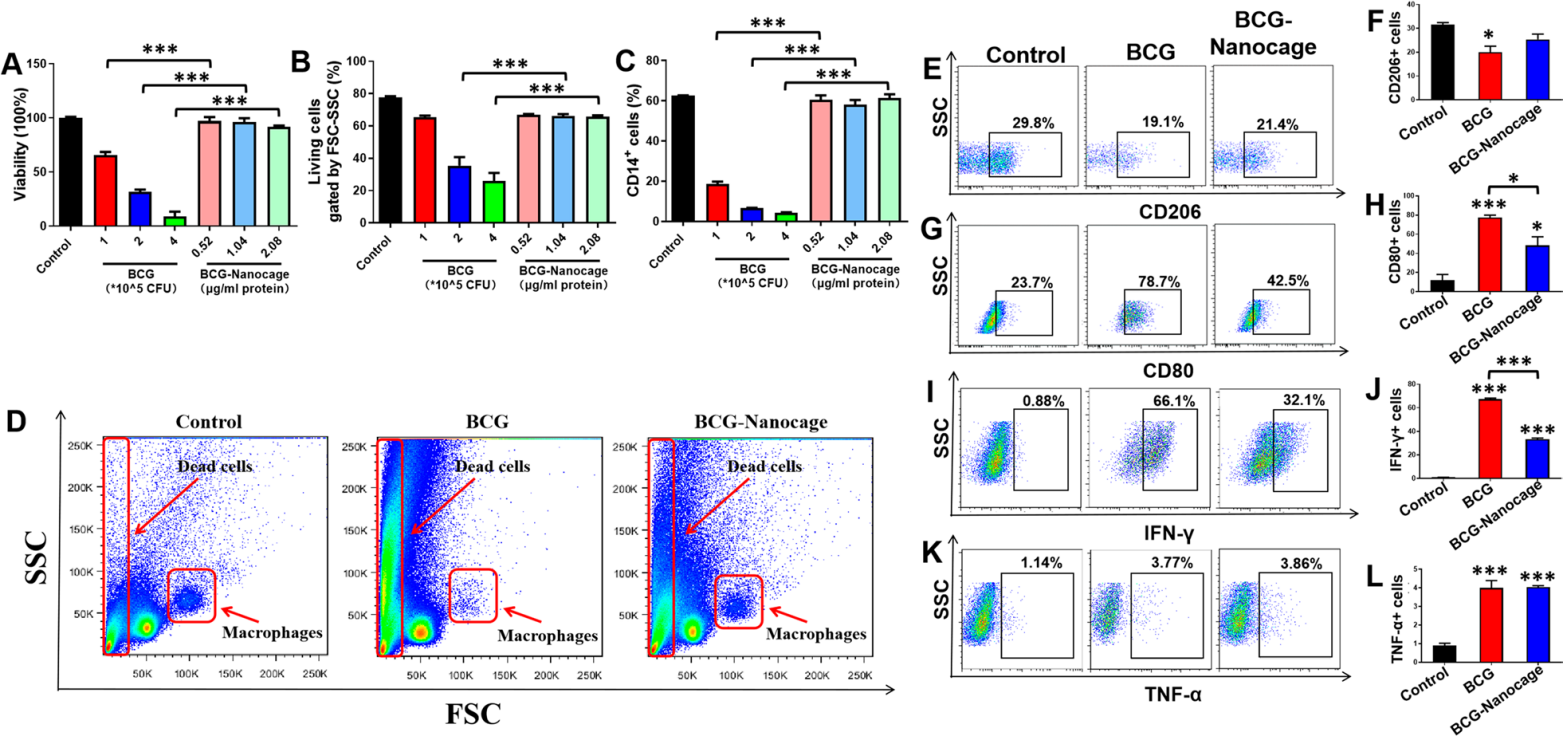
BCG-Nanocage promote antibacterial responses of macrophages (MΦ) with reduced cytotoxicity than live BCG bacillus. **A** Effects of live BCG bacillus and BCG-Nanocage on cell viability of THP-1 macrophages after 3 day treatment determined by MTT assay. **B** Effects of BCG and BCG-Nanocage on living cell percentages of THP-1 macrophages after 3 day treatment, followed by flow cytometry analysis. **C** Effects of BCG and BCG-Nanocage on CD14+ macrophages of THP-1 cells after 3 day treatment, followed by flow cytometry analysis. **D** Typical flow cytometry panel of BCG- or BCG-Nanocage-treated PBMCs from rhesus macaques after 6 day treatments. **E** Typical flow cytometry panel for the CD206 expression in THP-1 macrophages after 3 days of BCG or BCG-Nanocage treatments. **F** Effects of BCG and BCG-Nanocage on CD206 expression on macrophages, n = 3, *p < 0.05. **G** Typical flow cytometry panel for the CD80 expression in THP-1 macrophages after 3 days of BCG or BCG-Nanocage treatments. **H** Effects of BCG and BCG-Nanocage on CD80 expression on macrophages, n = 3, *p < 0.05, ***p < 0.001. **I** Typical flow cytometry panel for the IFN-γ expression in THP-1 macrophages after 3 days of BCG or BCG-Nanocage treatments. **J** Effects of BCG and BCG-Nanocage on IFN-γ+ macroghages, n = 3, ***p < 0.001. **K** Typical flow cytometry panel for the TNF-α expression in THP-1 macrophages after 3 days of BCG or BCG-Nanocage treatments. **L** Effects of BCG and BCG-Nanocage on TNF-α expression by macrophages, n = 3, ***p < 0.001

Since macrophages (MΦ) play a central role in mediating presentation of bacterial antigens and activation of T cells for mounting protective immunity against Mtb infection [38], we then evaluated the effects of BCG-Nanocage on preservation of viability and promotion of activation of macrophages. THP-1 macrophages were infected with live BCG or incubated with BCG-Nanocage with the same protein contents and then subjected to MTT (3-[4,5-dimethylthiazol-2-yl]-2,5 diphenyl tetrazolium bromide) and flow cytometry analysis to determine the viability of macrophages. The results indicated that live BCG induced significant viability inhibition in THP-1 cells while no significant viability inhibition was observed in BCG-Nanocage-treated THP-1 cells (Fig. 2A). The gating of live cells by flow cytomotry indicated significantly reduced percentages of live cells in THP-1 cells infected with live BCG in a dose-dependent manner, however, no significant viability inhibition was observed in BCG-Nanocage-treated THP-1 cells (Fig. 2B). Also, BCG infection also resulted in significant reduction of CD14+ cells in THP-1 cells, which was not observed in BCG-Nanocage-treated THP-1 cells (Fig. 2C).

Then, peripheral blood mononuclear cell (PBMC) from rhesus macaques were infected with live BCG or treated with BCG-Nanocage with the same protein contents for further flow cytometry analysis. While direct gating analysis of the flow map demonstrated the disappearance of monocyte population after BCG infection, BCG-Nanocage treatment didn’t induce similar disappearance of monocyte population (Fig. 2D). Additionally, the inhibition effects of BCG on CD14+ cells, CD3+ T cells, CD20+ B cells and CD56+ NK cells were also significantly reversed when treated with BCG-Nanocage with the same protein contents (Additional file 1: Fig. S4). These results indicated that BCG-Nanocage significantly reduced the cytotoxicity of BCG in vitro and ex vivo, and much lower cytotoxicity against macrophages might serve as a more effective machinery for antigen processing and presentation.

We then analyzed the properties of BCG-Nanocage to induce the activation of macrophages. THP-1 macrophages were incubated with BCG-Nanocage and live BCG bacteria, respectively, and BCG-Nanocage-incubated or BCG-infected macrophages were then subjected to analyses of the expression of activation/co-stimulation markers and production of pro-inflammatory cytokines. It is interesting to find that BCG-Nanocage show comparable levels of expression of activation/co-stimulation markers including CD206 (Fig. 2E–F) and CD80 (Fig. 2G–H) as well as pro-inflammatory cytokines including IFN-γ (Fig. 2I–J) and TNF-α (Fig. 2K–L). These results collectively suggest that BCG-Nanocage still well reserves the immune regulation effects of live BCG bacteria to induce macrophage activation, however, displays much lower cytotoxicity than live BCG bacteria.

### BCG-Nanocage, like live BCG bacilli, exhibited the robust capability to activate and expand innate-like Vγ2+ T effector cells, CD4+ T and CD8+ T effector cells of rhesus macaques in the PBMC culture

Since BCG-Nanocage reserve the immunological regulation effects of live BCG bacteria for macrophage (MΦ) activation, we then determined whether BCG-Nanocage still preserve the ability of live BCG bacteria to activate responses of T cells. We first focused on CD4+ /CD8+ αβ effector T cells to evaluate whether there were any beneficial effects conferred by micro-to-nano scale shift upon production of BCG-Nanocage from live BCG. To address this, PBMC derived from macaques were co-cultured with BCG-Nanocage or live BCG in presence of recombinant IL-2, followed by intracellular cytokine staining (ICS) and flow cytometric analyses for productions of anti-TB cytokines or cytotoxic molecules, such as IFN-γ, TNF-α, perforin, granzyme B, and granulysin. It is worth to note that BCG-Nanocage/IL2 co-stimulation could induce significant activation and expansion of CD4+ CD161+ T cells (Additional file 1: Fig. S5) and CD8+ T cells (Additional file 1: Fig. S6) with high expression levels of intracellular anti-TB cytokines/cytotoxic molecules, such as perforin, granzyme B and granulysin, and such activation and expansion of CD4 + CD161+ T effectors and CD8+ T effectors were comparable with co-stimulation with BCG/IL2 (Additional file 1: Fig. S5, S6). Thus, this data suggested that BCG-Nanocage maintain the ability of BCG to induce activation of CD4+ /CD8+ αβ effector T cells.

**Fig. 3:**
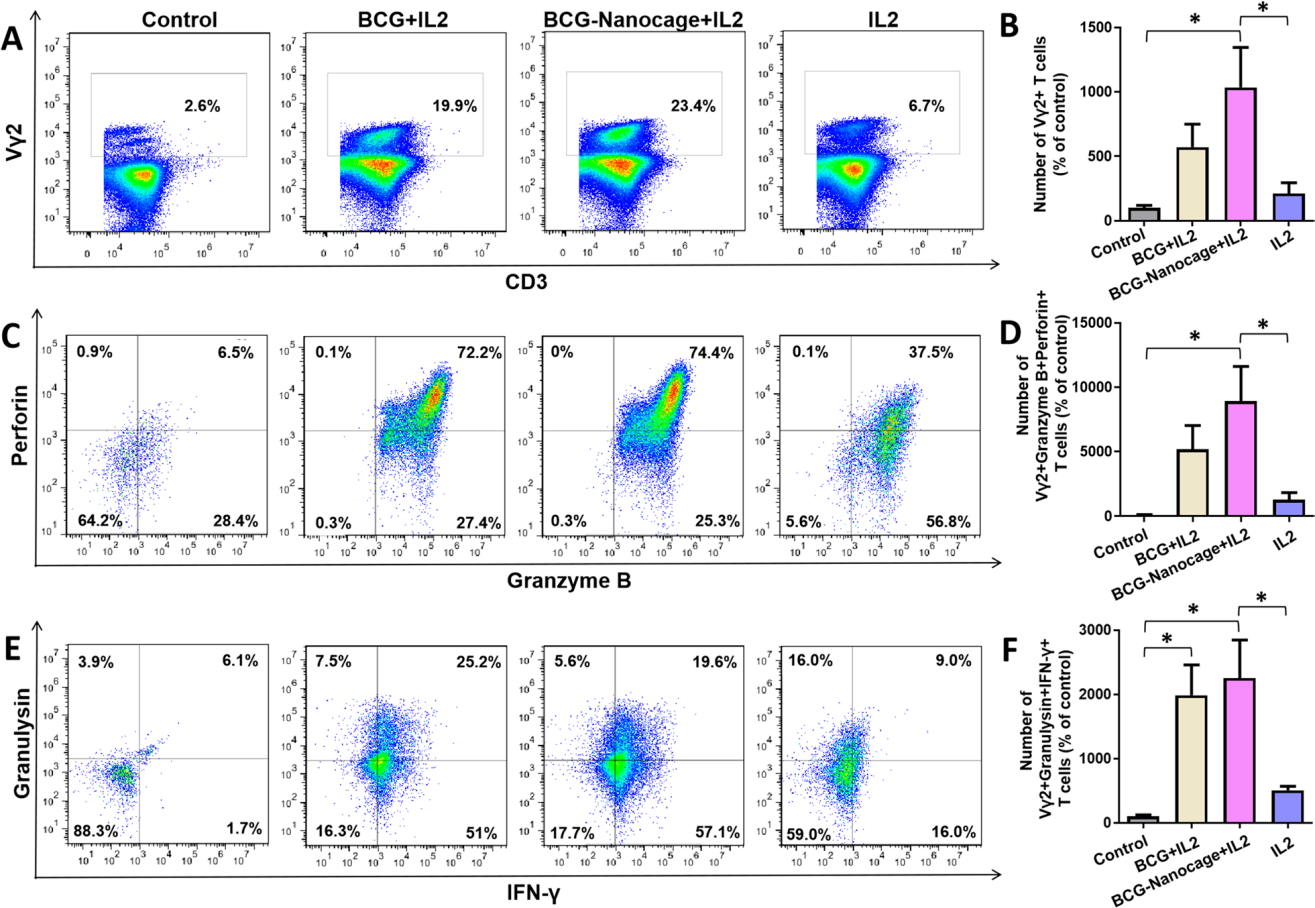
Effect BCG-Nanocage on e*x vivo* activation and expansion of Vγ2+ T cells in PBMC from non-human primates. **A** Typical flow cytometry panel for Vγ2+ cells gated in T cells from PBMC with or without (co-)stimulation using BCG/IL2, BCG-Nanocage/IL2 or IL2 for 6 days. **B** Effects of (co-)stimulation using BCG/IL2, BCG-Nanocage/IL2 or IL2 on Vγ2+ T cells after 6 day treatment, n = 3, *p < 0.05. **C** Typical flow cytometry panel for Granzyme B+ Perforin+ cells gated in Vγ2+ T cells in total T cells from PBMC with or without (co-)stimulation using BCG/IL2, BCG-Nanocage/IL2 or IL2 for 6 days. **D** Effects of (co-)stimulation using BCG/IL2, BCG-Nanocage/IL2 or IL2 on Vγ2+ Granzyme B+ Perforin+ cells in T cells after 6 day treatment, n = 3, *p < 0.05. **E** Typical flow cytometry panel for Granulysin + IFN-γ + cells gated in Vγ2+ T cells from PBMC with or without (co-)stimulation using BCG/IL2, BCG-Nanocage/IL2 or IL2 for 6 days. **F** Effects of (co-)stimulation using BCG/IL2, BCG-Nanocage/IL2 or IL2 on Vγ2+ Granulysin + IFN-γ+ cells in T cells after 6 day treatment, n = 3, *p < 0.05

In addition to the ability preservance of BCG-Nanocage to activate the CD4+ /CD8+ αβ effector T cells, we then evaluate whether shift of micro-to-nano scale upon production of BCG-Nanocage from live BCG would expand the spectrum of T cell immune responses. Vγ2Vδ2 T (also known as Vγ9Vδ2 T in humans) cells are a critical T cell subset existing in human and nonhuman primates that can be activated with metabolites from Mtb or BCG and mount protective immunity against Mtb infection [39]. We thus selected Vγ2 T cells as a prototype to determine whether and how such BCG-Nanocage could activate the Vγ2Vδ2 T cells. PBMC isolated from rhesus macaques were treated with BCG-Nanocage and live BCG bacteria, respectively, in presence of IL-2 co-stimulation. Similar with BCG/IL2 co-stimulation, BCG-Nanocage/IL2 co-stimulation could significantly expand Vγ2 T cells, and such Vγ2 T cell expansion effects were even stronger than BCG/IL2 co-stimulation (Fig. 3A–B). The numbers of multifunctional Granzyme B + Perforin + Vγ2 T effectors upon BCG-Nanocage/IL2 co-stimulation were also larger than those of BCG/IL2 co-stimulation (Fig. 3C–D), suggesting that BCG-Nanocage could induce Vγ2 T cells to produce more anti-TB protective cytokines or cytotoxic molecules. The numbers of Granulysin + IFN-γ + Vγ2 T effectors upon BCG-Nanocage/IL2 co-stimulation showed similar results with those of BCG/IL2 co-stimulation (Fig. 3E–F), further suggested the reserved ability of BCG-Nanocage to induce anti-TB protective cytokines/cytotoxic molecules production in Vγ2 T cells. Thus, these results collectively suggested that BCG-Nanocage induced similar levels of anti-TB effector functions of CD4+ and CD8+ αβ T cells, which is comparable with live BCG bacteria, and induced stronger Vγ2Vδ2 T cell responses than live BCG bacteria did.

### BCG-Nanocage immunization of rhesus macaques elicited similar or stronger immune responses of Vγ2Vδ2 T cells as well as Vγ2Vδ2 T and CD4+ /CD8+ T effectors compared to live BCG vaccination

**Fig. 4:**
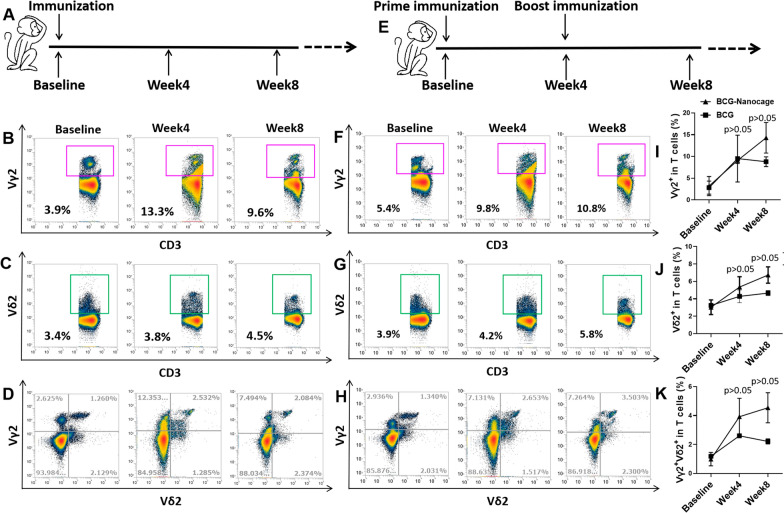
BCG-Nanocage show great potential in priming and boosting Vγ2Vδ2 T cells in non-human primates. **A** Schematic diagram of BCG immunization in rhesus macaques. Typical flow images for (**B**) Vγ2+ T cell, **C** Vδ2+ T cell and **D** Vγ2 + Vδ2+ T cell in T cells from PBMC of rhesus macaques before and after BCG immunization. **E** Schematic diagram of BCG-Nanocage boost immunization in rhesus macaques. Typical flow images for (**F**) Vγ2+ T cell, **G** Vδ2+ T cell and **H** Vγ2 + Vδ2+ T cell in T cells from PBMC of rhesus macaques before and after BCG-Nanocage immunization. **I** Vγ2+ T cell, **J** Vδ2+ T cell and **K** Vγ2 + Vδ2+ T cell in T cells from PBMC of rhesus macaques before and after BCG immunization or BCG-Nanocage immunization

We then determined the potential in vivo immunization advantages of BCG-Nanocage. We used our expertise of nonhuman primate model of TB infection and BCG vaccinations [27–29, 31] to evaluate the effects of BCG-Nanocage immunization on Vγ2Vδ2 T cell responses. Rhesus macaques were first primed using subcutaneous treatments with BCG-nanocage, followed by boost at week 4 (Fig. 4E), and the typical immunization method of BCG was served as a control (Fig. 4A). Standard physical follow-ups, complete blood counts and other routine lab parameters indicated that BCG-Nanocage immunizations were safe, without any apparent local or systemic side effects. PBMC of macaques were then collected for analyses of numbers and effector functions of T cells at baseline and following immunization. It is shown that the immunization with BCG could induce the expansion of Vγ2 T cells, Vδ2 T cells and Vγ2Vδ2 T cells in the PBMC of rhesus macaques in 4 weeks, however, would undergo partially retracement in 8 weeks (Fig. 4B–D and I–K). Interestingly, prime immunization using BCG-Nanocage induce more significant expansion of Vγ2 T cells (Fig. 4F and I), Vδ2 T cells (Fig. 4G and J) and Vγ2Vδ2 T cells (Fig. 4H and K) in peripheral blood of macaques in 4 weeks than that of BCG. Boost vaccination of BCG-Nanocage in BCG-Nanocage-primed macaques could further enhance the expansion of Vγ2 T cells (Fig. 4F and I), Vδ2 T cells (Fig. 4G and J) and Vγ2Vδ2 T cells (Fig. 4H and K) in the peripheral blood of rhesus macaques. These results indicated that BCG-Nanocage immunization soundly induce in vivo expansions and immune memory of Vγ2Vδ2 T cells.

**Fig. 5:**
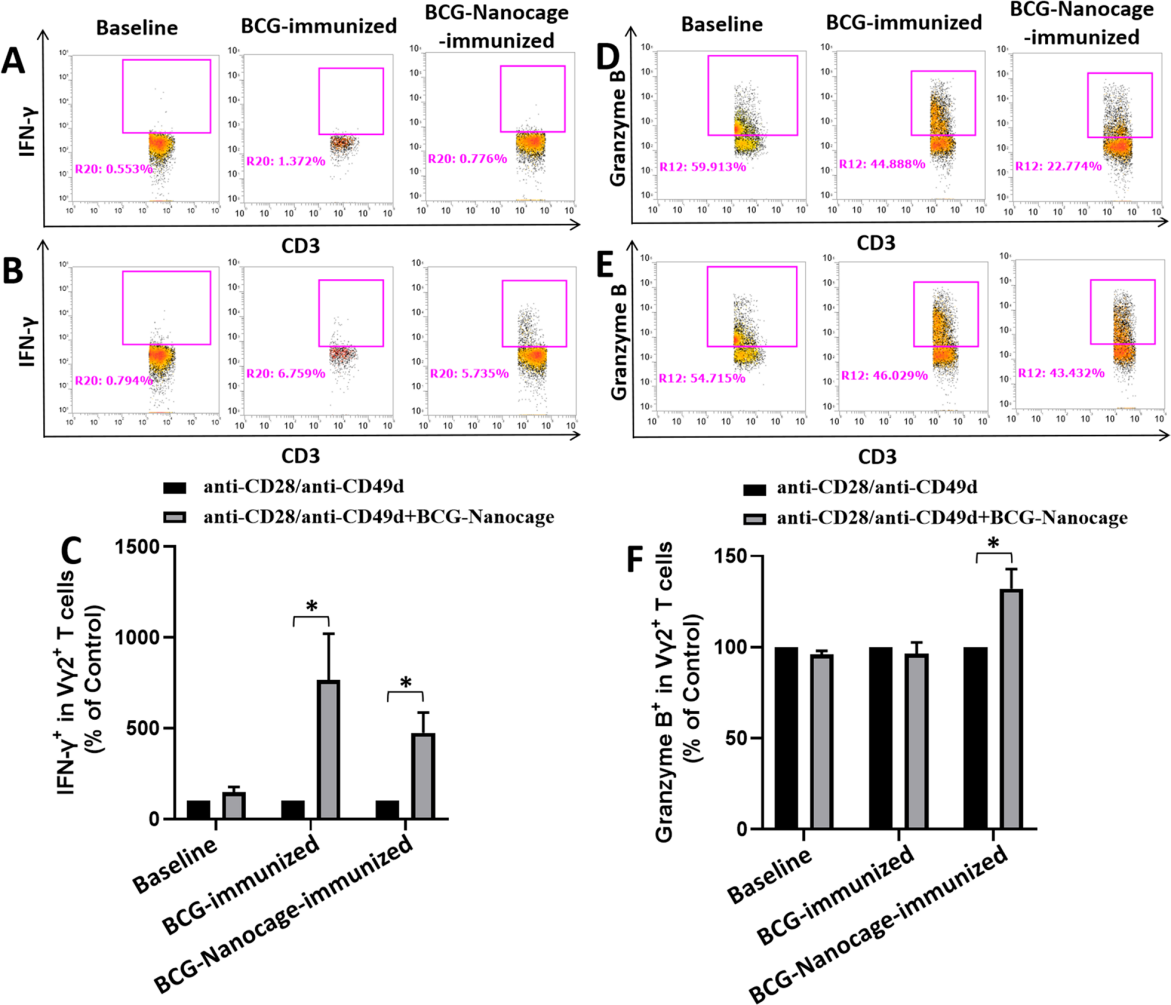
BCG-Nanocage boost immunization in non-human primates enhance IFN-γ and Granzyme B production in Vγ2Vδ2 T cells. Typical flow images for intracellular IFN-γ level in Vγ2+ T cells from PBMC of rhesus macaques before and after BCG immunization or BCG-Nanocage immunization with (**A**) anti-CD28/anti-CD49d stimulation or (**B**) co-stimulation with anti-CD28/anti-CD49d and BCG-Nanocage, cells were gated in CD3 + Vγ2 + cells. **C** Statistical results for co-stimulation using anti-CD28/anti-CD49d and BCG-Nanocage induced changes of intracellular IFN-γ levels in Vγ2+ T cells from PBMC of rhesus macaques before and after BCG or BCG-Nanocage immunization, data were expressed as percentages of control, n = 4, *p < 0.05. Typical flow images for intracellular level of Granzyme B in Vγ2+ T cells from PBMC of rhesus macaques before and after BCG or BCG-Nanocage immunization with (**D**) anti-CD28/anti-CD49d stimulation or (**E**) co-stimulation anti-CD28/ anti-CD49d and BCG-Nanocage, cells were gated in CD3+ Vγ2+ cells. **F** Statistical results for co-stimulation using anti-CD28/anti-CD49d and BCG-Nanocage induced changes of intracellular Granzyme B levels in Vγ2+ T cells from PBMC of rhesus macaques before and after BCG or BCG-Nanocage immunization, data were expressed as percentages of control, n = 4, *p < 0.05

To determine the effects of BCG-Nanocage on T cell effector functions, PBMC derived from macaques at baseline (before immunization), macaques immunized with live BCG bacteria or macaques immunized with BCG-Nanocage, were ex vivo re-stimulated using (i): anti-CD28 + anti-CD49d or (ii): anti-CD28 + anti-CD49d plus BCG-Nanocage. The ex vivo re-stimulation using anti-CD28 + anti-CD49d would further enhance the production of effector molecules through T cell co-stimulation signaling, and ex vivo stimulation using BCG-Nanocage would allow us detect the memory or recall response of T cells that used to engage with BCG-Nanocage in macaques. The stimulated PBMC were then subjected for intracellular staining of effector molecules including IFN-γ and granzyme B and flow cytometric analysis. BCG-Nanocage-immunized macaques showed significantly higher levels of IFN-γ and granzyme B production by Vγ2Vδ2 T cells than that of baseline samples (Fig. 5). Vγ2Vδ2 T cells in BCG-Nanocage immunized and BCG-vaccinated macaques showed appreciable ability to produce IFN-γ (Fig. 5A–C), suggesting the excellent immune memory of Vγ2Vδ2 T cells induced by BCG-Nanocage treatments. More importantly, granzyme B production in PBMC from BCG-Nanocage-immunized macaques was significantly higher than that of BCG-immunized macaques (Fig. 5D–F). This data suggested that BCG-Nanocage immunization was more potent to stimulate immune memory for cytolytic effector functions of Vγ2Vδ2 T cells than live BCG bacteria immunization did.

Additionally, we also evaluated whether BCG-Nanocage immunization could induce expression of cytotoxic molecules by CD4+ and CD8+ T cells. It is shown that ex vivo re-stimulation using anti-CD28 + anti-CD49d plus BCG-Nanocage induced significantly higher intracellular IFN-γ levels in CD4+ and CD8+ T cells in PBMC derived from rhesus macaques immunized with BCG or BCG-Nanocage (Additional file 1: Fig. S7). More interestingly, ex vivo re-stimulation using anti-CD28+ anti-CD49d plus BCG-Nanocage only increased the intracellular Granzyme B levels in CD4+ and CD8+ T cells from macaques immunized with BCG-Nanocage (Additional file 1: Fig. S8) but not in baseline macaques or BCG-immunized macaques. These results indicated that BCG-Nanocage immunization could also enhance the ability of CD4+ and CD8+ T cells to produce anti-TB cytokines or cytolytic molecules, further highlighting the potency of BCG-Nanocage to enhance Th1/Th1-like or cytotoxic responses of CD4+ or CD8+ T cells.

### BCG-Nanocage-immunized macaques developed rapidly-sustained pulmonary responses of Vγ2Vδ2+ T cells upon Mtb challenge

**Fig. 6:**
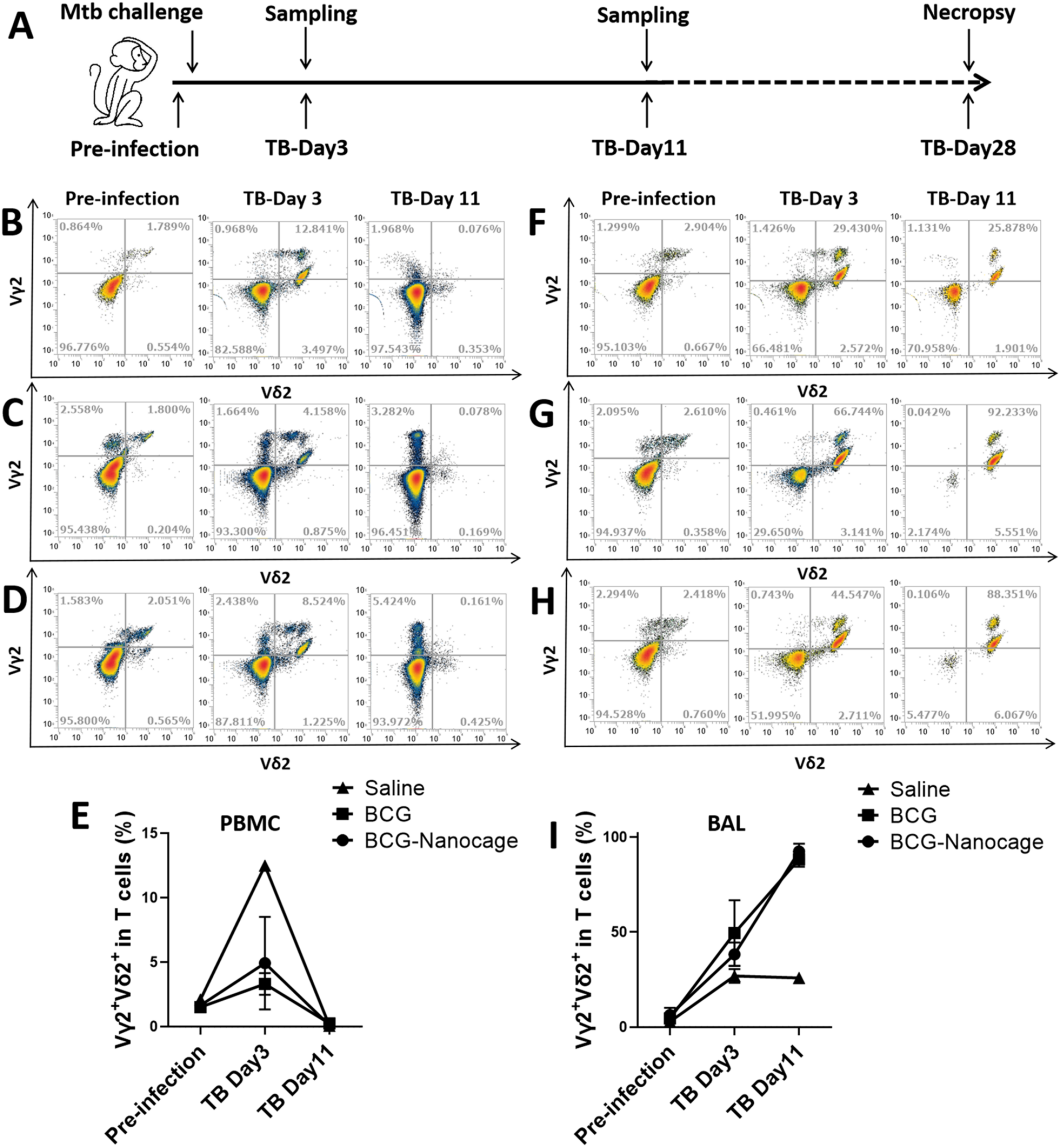
BCG-Nanocage immunization enhance pulmonary Vγ2+ Vδ2+ T cell responses in non-human primates upon Mtb challenge. **A** Schematic diagram of Mtb challenge in rhesus macaques. Typical flow images for Vγ2+ Vδ2+ T cell level in T cells in PBMC from (**B**) saline, **C** BCG-immunized and **D** BCG-Nanocage-immunized rhesus macaques before and after Mtb infection. **E** Changes of Vγ2+ Vδ2+ T cell level in T cells in PBMC from saline, BCG-immunized and BCG-Nanocage-immunized rhesus macaques before and after Mtb infection, for saline treated macaques, ^p < 0.05,^^p < 0.01, for BCG vaccined macaques, #p < 0.05, for BCG-Nanocage vaccined macaques, *p < 0.05. Typical flow images for Vγ2+ Vδ2+ T cell level in T cells in bronchoalveolar lavage (BAL) from (**F**) saline, **G** BCG-immunized and **H** BCG-Nanocage-immunized rhesus macaques before and after Mtb infection. **I** Changes of Vγ2+ Vδ2+ T cell level in T cells in BAL from saline, BCG-immunized and BCG-Nanocage-immunized rhesus macaques before and after Mtb infection, for saline treated macaques, ^p < 0.05, for BCG vaccined macaques, #p < 0.05, ##p < 0.01, for BCG-Nanocage vaccined macaques, *p < 0.05, **p < 0.01

Since BCG-Nanocage immunization induce strong immune memory responses of Vγ2Vδ2 T cells in macaques, we then analyzed whether such immune responses of Vγ2Vδ2 T cells with BCG-Nanocage immunization could also be observed upon Mtb challenge. BCG-Nanocage-immunized macaques, BCG-immunized macaques and saline-treated control macaques were then challenged with a low colony forming unit (CFU) dosage of Mtb (5 CFU of H37Rv) at right caudal lobe of lungs, and samples were collected for analysis at designed times (Fig. 6A). Compared with saline-treated macaques, Vγ2+ Vδ2+ T cells in PBMC rapidly increased after 3 days of Mtb infection in BCG or BCG-Nanocage-immunized macaques (Fig. 6B–E). Particularly, Vγ2+ Vδ2+ T cells rapidly increased in the bronchoalveolar lavage (BAL) after 3 days of Mtb infection, and percentages of Vγ2+ Vδ2+ T cells underwent approximately Two to  threefold further increase after 11 days infection in BCG- or BCG-Nanocage- immunized macaques, but not in saline-treated controls (Fig. 6F–I). However, Vγ2+ Vδ2+ T cells in PBMC rapidly decreased after 11 days of infection (Fig. 6B–E), which might be attributed to the migration or accumulation of peripheral Vγ2+ Vδ2+ T cells into infection sites in lungs. Notably, the fast-acting accumulation of Vγ2+ Vδ2+ T cells in the lung compartment of BCG- or BCG-Nanocage-immunized macaques after Mtb challenge would allow Vγ2+ Vδ2+ T cells to mount anti-TB immunity [27, 29].

**Fig. 7:**
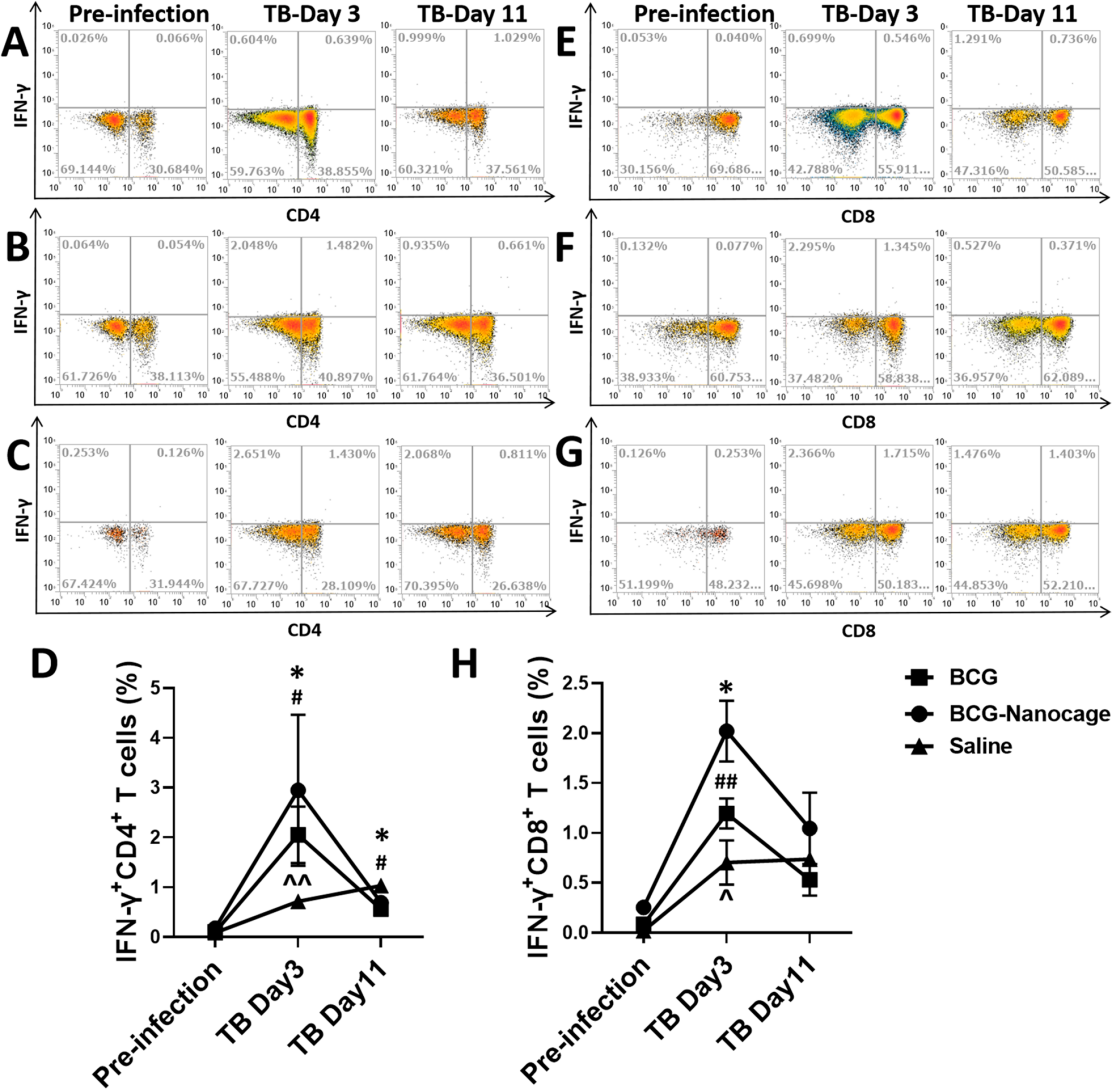
BCG-Nanocage immunization induce rapid pulmonary Th1/Th1-like responses in non-human primates upon Mtb challenge. Typical flow images for IFN-γ + CD4 + Th1 effector cells in the T cells of bronchoalveolar lavage (BAL) from (**A**) saline, **B** BCG-immunized and **C** BCG-Nanocage-immunized rhesus macaques before and after Mtb infection. **D** Changes of IFN-γ+ CD4+ Th1 effector cells in the T cells of bronchoalveolar lavage (BAL) from saline, BCG-immunized and BCG-Nanocage-immunized rhesus macaques before and after Mtb infection, for saline treated macaques, ^^p < 0.01, for BCG vaccined macaques, #p < 0.05, for BCG-Nanocage vaccined macaques, *p < 0.05. Typical flow images for IFN-γ+ CD8+ Th1-like effector cells in T cells in BAL from (**E**) saline, **F** BCG-immunized and **G** BCG-Nanocage-immunized rhesus macaques before and after Mtb infection. **H** Changes of IFN-γ+ CD8+ Th1-like effector cells in T cells in BAL from saline, BCG-immunized and BCG-Nanocage-immunized rhesus macaques before and after Mtb infection, for saline treated macaques, ^p < 0.05, for BCG vaccined macaques, ##p < 0.01, for BCG-Nanocage vaccined macaques, *p < 0.05

We also evaluated the responses of IFN-γ+ CD4+ T cells and IFN-γ+ CD8+ T cells in the PBMC and bronchoalveolar lavage (BAL) of rhesus macaques after Mtb challenge. Although the levels of IFN-γ+ CD4+ cells and IFN-γ+ CD8+ cells in PBMC decreased slightly in all macaques after Mtb infection (Additional file 1: Fig. S9), IFN-γ+ CD4+ cells and IFN-γ+ CD8+ cells in bronchoalveolar lavage (BAL) increased more rapidly and significantly in BCG and BCG-Nanocage immunized macaques than that of saline-treated macaques after 3 days of Mtb infection (Fig. 7). The increased IFN-γ+ CD4+ cells and IFN-γ+ CD8+ cells in bronchoalveolar lavage (BAL) of BCG- or BCG-Nanocage immunized macaques in Day 3 then decreased to the similar level with saline-treated macaques after 11 days of Mtb infection (Fig. 7). This data suggested that, compared with BCG immunization, BCG-Nanocage immunization induced similar levels of responses of IFN-γ+ CD4+ T cells and IFN-γ+ CD8+ T cells in the lungs of rhesus macaques upon Mtb challenge. Taken together, these pieces of data suggested that BCG-Nanocage immunization induced fast-acting immune responses of Vγ2+ Vδ2+ T cells and appreciable levels of IFN-γ+ CD4+ T cell and IFN-γ+ CD8+ T cell responses in the lungs during Mtb infection in macaques.

### BCG and BCG Nanocage immunized macaques, but not saline controls, exhibited undetectable Mtb infection loads or TB lesions in the Mtb-challenged lung lobe and hilar LN at endpoint after challenge

**Fig. 8:**
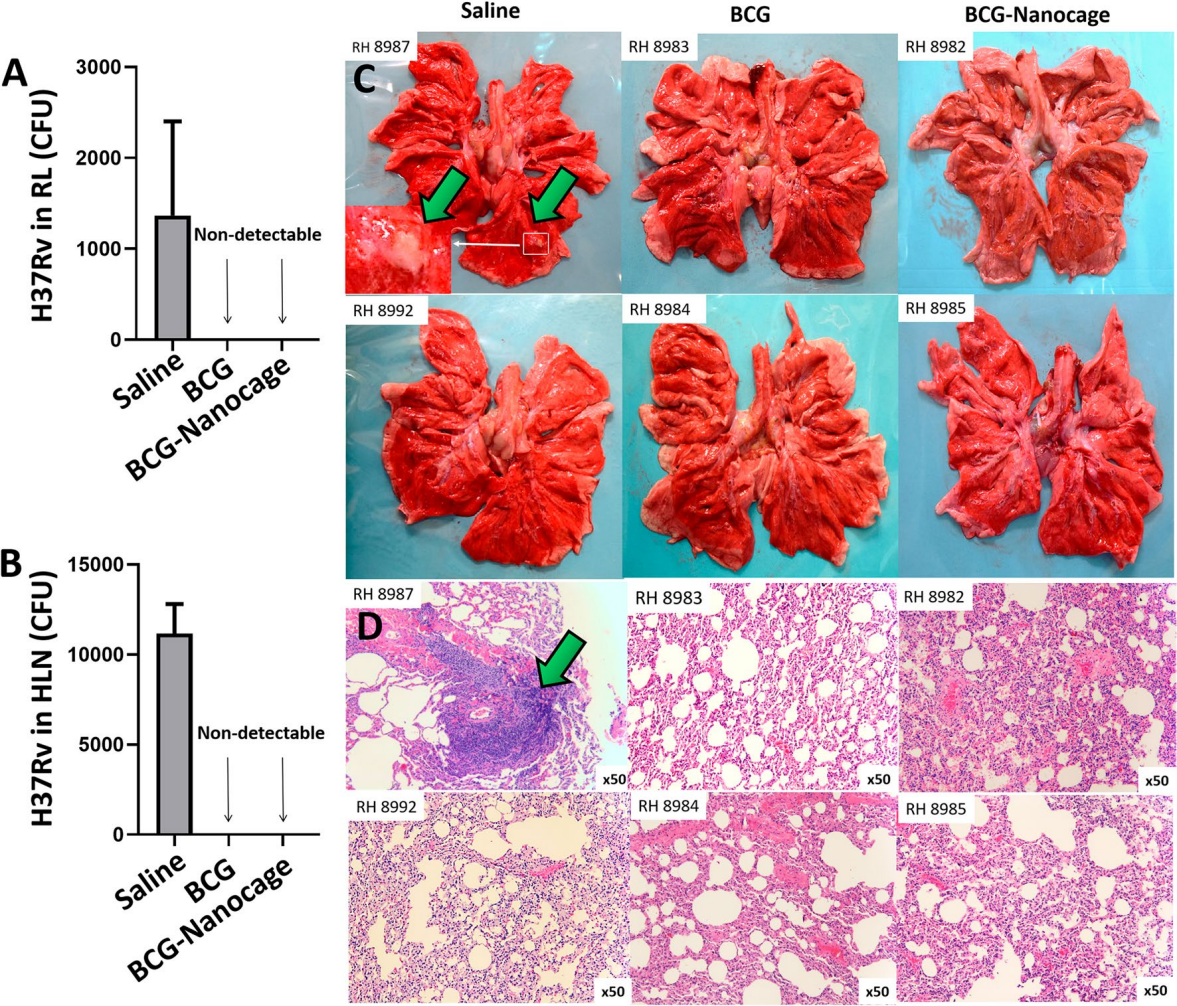
BCG-Nanocage immunization confer immune protection against Mtb challenge and alleviate pathological damages in lungs in Mtb-infected non-human primates. **A** CFU of H37Rv in the right-lower lobes of rhesus macaques. **B** CFU of H37Rv in the hilar lymph node (HLN) of rhesus macaques. **C** Typical granuloma observed in the lung of saline-treated but not in BCG- immunized or BCG-Nanocage-immunized rhesus macaques after Mtb challenge. Note that large-scale necrotic granuloma was observed in right lobes of RH #8987 macaques, green arrows indicated the necrotic granuloma. **D** Typical hematoxylin and eosin (H&E) staining of right lobes of macaques from indicated groups. Note that large-scale necrotic granuloma was observed in right lobes of RH #8987 macaques, green arrows indicated the necrotic granuloma

We then evaluated whether such BCG-Nanocage immunization was associated with immune protection against Mtb challenge, and Mtb-infected macaques were subjected to analyses of Mtb burdens and TB pathology. Quantitative analyses based on colony forming unit (CFU) counting of Mtb H37Rv were applied to determine the Mtb burdens. BCG- or BCG-Nanocage-immunized macaques showed undetectable H37Rv CFU counts (Fig. 8A) in the right caudal lung lobe, indicating the clearance of H37Rv in the right caudal lung lobe of BCG- or BCG-Nanocage-immunized macaques. There were also no H37Rv CFU counts were observed in the hilar lymph node (HLN) of BCG- or BCG-Nanocage-immunized macaques (Fig. 8B). These data suggested that, compared with saline control and live BCG, BCG-Nanocage conferred much better immune sterilizations of pulmonary Mtb burdens.

Analyses of lung gross pathology (Fig. 8C) and hematoxylin–eosin (HE)-stained lung sections (Fig. 8D) indicated typical necrotic granulomas in the right caudal lung lobe of saline-treated macaques. However, no granuloma like structures were observed in the right caudal lung lobe of BCG- or BCG-Nanocage-immunized macaques (Fig. 8C–D), indicating the protective effects of BCG- or BCG-Nanocage immunization in nonhuman primates. Moreover, we found significant swelling and necrotic granuloma-like structures in the hilar lymph nodes of saline treated macaques (Additional file 1: Fig. S10). In contrast, no significant hilar lymph node swelling or necrotic granuloma-like structures were found in BCG- or BCG-Nanocage- immunized macaques (Additional file 1: Fig. S10). These results collectively indicate that BCG and BCG-Nanocage immunizations mount similar immune protections against TB lung and hilar lymph node pathology.

## Discussion

Although the efficacy of childhood *Bacillus Calmette-Guerin* (BCG) vaccination in the prevention of adult pulmonary tuberculosis is not universally accepted, there are also some evidences support the efficacy of BCG vaccination in the prevention of adult pulmonary TB disease [40]. The concept of using BCG for intravenous vaccination has been proved to be effective to protect non-human primates from Mtb infection [13, 41], which reflect the potentials of changing BCG vaccination routes for more effective anti-TB vaccination beyond the typical intracutaneous vaccination. However, intravenous injection is still not accepted for vaccine administration due to the uncontrollable immunological responses when vaccine direct expose to the large amounts of immune cells in blood. And the intravenous administration of live BCG might also raise a greater safety concern about the wide-spread BCG dissemination, which therefore requires us to develop novel vaccines that can be used for subcutaneous administration or intramuscular administration to activate more potent and controllable immunological responses with few side effects. In this work, we introduce a strategy to engineer BCG into functional BCG-Nanocage, which is expected to induce potent anti-TB protective immunity of T cell subsets with enhanced safety for potential anti-TB vaccination. We further applied these BCG-Nanocage for subcutaneous vaccination in nonhuman primates using the typical intracutaneous vaccination of BCG as control. Standard physical follow-ups and complete blood counts and other routine lab parameters throughout the experiments indicated that BCG-Nanocage immunizations were safe, without any apparent local or systemic side effects.

BCG-nanocage induce broad-spectrum immune responses of anti-TB protective T-cell subsets, including Th1/Th1-like cells, CD8+ cytotoxic cells, as well as Vγ2Vδ2 T cells, and such broad immune response induced by BCG-Nanocage immunization are associated with effective immune protection against Mtb infection and TB pathology in Mtb-infected nonhuman primates. Notably, compared with live BCG, BCG-Nanocage induced similar levels of immune responses of Th1/Th1-like cells and CD8+ cytotoxic cells but much stronger Vγ2Vδ2 T cell immune responses. Such successful induction of immune responses Vγ2Vδ2 T cells, Th1/Th1-like cells and CD8+ cytotoxic cells was associated with immune protection against Mtb infection and pulmonary pathology in Mtb-infected macaques. Thus, BCG-Nanocage technology not only largely preserve the immune activity of live BCG but also confer more potent or much broader immune responses of protective T cell subsets to confer better protection against deadly Mtb infection. Due to the lack of nonhuman primates for researches during Covid-2019 epidemic, it was a pity that only two macaques in each group were used to preliminarily prove the potentials of BCG-Nanocage for anti-TB vaccination. Our initial anti-TB function studies of BCG-Nanocage in nonhuman primates strongly suggested their potentials as protective anti-TB vaccine candidate, which requires further larger-scale nonhuman primate studies as pre-clinical evidences.

The mechanisms underlying the stronger or broader T cell immune responses induced by BCG-Nanocage immunization in macaques may be multiple folds: (i) The shift of microscale live BCG with clumped-clustered features to a more uniform, nanoscale particles would allow macrophage(MΦ) more readily to uptake and process the BCG-Nanocage; (ii) The BCG-Nanocage may serve as a nanoparticle carrying the antigens to facilitate the delivery of antigens into antigen processing and presentation machinery involving in phagosome- lysosome system. Such nanoparticle-enhanced delivery of drug molecules into phagosome- lysosome system of macrophage(MΦ) have been recently demonstrated by us [22]; (iii) Compared with infection with live BCG, the immunization with the much safer BCG-Nanocage could reduce the death of macrophage (antigen presentation cells), which would theoretically allow better processing and presentation of antigens by macrophages(MΦ) to T cells. However, the exact mechanisms underlying how BCG-Nanocage immunization induce broader or more potent T cell immune responses, particularly Vγ2Vδ2 T cells, remain further studies. Additionally, as alveolar macrophages are the main target cells for Mtb and play very important roles in anti-TB immunity, we therefore choose macrophage as a model of APCs to explore the effects of BCG-Nanocage in APCs for our current study. It’s true that DCs also play critical roles in Mtb infection and anti-TB immunity, which is also very important for BCG-Nanocage induced adaptive immune responses. To demonstrate more clear illustration of BCG-Nanocage induced anti-TB effects, we would also explore the effects of BCG-Nanocage on DCs in our future works, which would be more helpful to demonstrate the anti-TB immunological effects of BCG-Nanocage.

γδ T cells are highly potent effector cells that straddling the innate and adaptive arms of the immune system in response to pathogens and tumors [42, 43]. Vγ2Vδ2 T cells existing only in human and nonhuman primates, account for the majority of circulating γδ T cells that providing fast-acting, multi-functional protection during infections [39]. Our results demonstrated that BCG-Nanocage and IL2 co-stimulation induced strong Vγ2Vδ2 T cell responses. This indicated that BCG-Nanocage not only preserved the ability of BCG to induce responses of Th1/Th1-like cells and CD8+ cytotoxic cells but also make the anti-TB immune responses more potent or broader through engineering BCG-Nanocage from clumped-clustered features BCG to more uniform nanoparticles. We have previously demonstrated the rapid and sustained increases of Vγ2Vδ2 T cells in the lungs of macaques vaccinated with HMBPP (4-hydroxy-3-methyl- 2-butenyl pyrophosphate)-producing listeria after Mtb challenge contributed to the inhibited intracellular Mtb growth and reduced pathology in the lungs and other organs [29]. Our previous works have also systemically and clearly demonstrated that the phosphoantigen- activated Vγ2Vδ2 T cells or adaptive transfer of Vγ2Vδ2 T cells can introduce protection effects against Mtb infection and TB pathology in nonhuman primates [27, 29, 30, 44].

Here, BCG-Nanocage-immunized macaques developed memory-like immune responses of Vγ2Vδ2 T cells that can be rapidly activated and exertion of effector functions of production of cytokines or cytotoxic molecules after ex vivo BCG-Nanocage stimulation or upon in vivo Mtb infection. With the rapid Vγ2Vδ2 T cell responses, macaques immunized with BCG-Nanocage demonstrated similar effects in lungs and hilar lymph nodes as like BCG to limit Mtb growth. This suggested that BCG-Nanocage could provide highly potent protection against Mtb infection and TB pathology in nonhuman primates via targeting Vγ2Vδ2 T cell activation and effector functions.

Th1 cells are a lineage of CD4+ effector T cell that produce IFN-γ, IL-2 and TNF-β to promote cell-mediated immune responses, such as activating macrophages and differentiating CD8+ T cells into cytotoxic lymphocytes (CTLs), which are critical for host defense against intracellular bacterial pathogens [45, 46]. Our results also indicated that BCG-Nanocage immunization could also promote expansions of Th1-like T cells against ex vivo pathogenic components stimulation and in vivo Mtb infection. Thus, BCG-Nanocage might also serve as a potential vaccine or adjuvants to enhance Th1/Th1-like T cell responses against Mtb infections.

Although development of BCG-Nanocage from live BCG appear to induce highly potent responses of Th1/Th1-like cells, CD8+ cytotoxic cells and Vγ2Vδ2 T cells in macaques, one feature for BCG-Nanocage is that BCG-Nanocage have lost some bacterial components of live BCG. By now, it remains unclear what and how much bacterial components were lost during the productions of BCG-Nanocage. As we widely known, during the immunogenicity process of typical BCG vaccination, BCG is phagocytosed by host cells (such as macrophages and dentritic cells) and proceed to the lysosomes for cell wall lysis by lysozome with the assistance of some hydrolases and the low pH environment. These processes are critical for the immunogenicity of BCG vaccination, contributing to the following antigen presentation by major histocompatibility complex (MHC) proteins to activate adaptive immunity. In the engineering of BCG-Nanocage, we treated BCG with EDTA and β-mercaptoethanol for lipopolysaccharide removal and disulfide bond destruction of outer membrane proteins. Then, lysozyme was applied to further catalyze the destruction of BCG cell wall by cleaving the peptidoglycan components and removing periplasmic components. These processes directly result in the partial loss of BCG cell wall components, which is also very important to stimulate inflammatory responses through the activation of different pattern recognition receptors (PRRs) [47]. Thus, the immunogenicity of BCG-Nanocage is partly attenuated during the preparation processes, however, the partial removal of the obstinated BCG cell membrane provides new possibilities making the intracellular BCG antigens more readily to be acquired and presented for adaptive immunity activation.

Interestingly, our data suggested that BCG-Nanocage still showed potent activation, expansion and exertion of effector functions of Vγ2Vδ2 T cells in nonhuman primates. This suggested that the critical antigens required to activate Vγ2Vδ2 T cells in BCG were well reserved during BCG-Nanocage preparation, or, these partially lost antigens to activate Vγ2Vδ2 T cells in BCG could be rescued by the size effects of BCG-Nanocage for its superior ability for antigen recognition, processing and presentation. In this work, BCG was selected as a control for BCG-Nanocage because BCG is still the most widely accepted TB vaccine worldwide and its high immunogenicity makes it more appropriate to act as a control. However, it’s true that the addition of a BCG protoplast group would be more persuasive to indicate the potential superiority for a BCG nano-vaccine engineered from BCG protoplast. Further comparisons about the immunological regulation effects between BCG-Nanocage and BCG protoplast would be very helpful to demonstrate the advantages of nanotechnique engineering, which would benefit the future nano-vaccine development.

Due to the lack of more effective TB vaccines, BCG vaccination is given to new born babies as vaccine to protect them from getting TB, especially TB meningitis, in a lot of countries. The failure of BCG to protect adults in some populations—in particular in some studies in India [48, 49], has always been used to criticize the useless of BCG in protecting adult from TB. Here, we proved that BCG-Nanocage vaccination could protect young adult rhesus macaques (4–8 years old) from small dosage of Mtb infection, which was expected to show potential protective effects for human adult vaccination. However, our results only showed the short-term protective effects of BCG-Nanocage vaccination against Mtb infection in macaques, without the long-term protective data in nonhuman primates. Thus, the research progress of our propose using BCG-Nanocage as TB vaccine candidate is still far from the clinical trials, which not only urges us to further explore the long-term protective effects of BCG-Nanocage vaccination in newborn nonhuman primates in the future works, but also requires us to test their potential protective effects against Mtb infection on adult nonhuman primates.

## Conclusions

Here, we have developed BCG-Nanocage as a highly potent and safe anti-TB immune protection system by taking advantages of high immunogenicity of *Bacillus Calmette-Guerin* (BCG) and the size/shape-shift effect conferred from microscale BCG with clumped-clustered features to nanoscale BCG-Nanocage. BCG-Nanocage demonstrated specific macrophage-targeting effects, well reserved macrophage activation effects, and reduced cytotoxicity on macrophage, which may therefore provide more potent antigen recognition, processing and presentation for mounting protective T cell immunity with increased safety. BCG-Nanocage immunization enhanced immune responses of Vγ2Vδ2 T cells, Th1/Th1-like cells and CD8+ cytotoxic T cells and protect macaques from a small dosage of Mtb infection with reduced Mtb growth and alleviated pathology in multiple organs such as lungs and hilar lymph nodes. This work highlights BCG-Nanocage as a novel immunization strategy to facilitate the development of more effective vaccines and therapeutics against deadly TB infection.

## Supplementary Information

**Additional file 1: Fig. S1.** Size of BCG-Nanocage upon -80℃storage. Size distribution of BCG-Nanocage before and after 70 days storage at −80℃, samples were thawed at room temperature for size measurements. **Fig. S2.** BCG preferentially target macrophages (MΦ), but not T cells, B cells or endothelium ex vivo. (A) Confocal imaging for cellular uptake of GFP-BCG by macrophages (MΦ) from the freshly isolated bronchoalveolar lavage (BAL) fluid of rhesus macaques after 1 h treatment of GFP-BCG, followed with anti-human CD11b-APC antibody and anti-human CD3-PE antibody staining, scale bar: 20 μm. (B) Flow cytometry analysis for cellular uptake of GFP-BCG by macrophages, T cells, B cells and endothelium from the freshly isolated intraepithelium (IEL) of rhesus macaques after 1 h treatment. (C) Cellular uptake of GFP-BCG by macrophages, T cells, B cells and endothelium from the freshly isolated intraepithelial lymphocytes of rhesus macaques after 1 h treatment, n = 4, ***p < 0.001. **Fig. S3.** Comparison of BCG and BCG-Nanocage uptake by macrophages (MΦ), T cells, B cells and endothelium ex vivo. Cellular uptake of GFP-BCG or GFP-BCG-Nanocage by macrophages, T cells, B cells and endothelium from the freshly isolated intraepithelial lymphocytes of rhesus macaques after 1 h treatment, n = 4. Cellular uptake of GFP-BCG or GFP-BCG-Nanocage by macrophages, T cells, B cells and endothelium from the freshly isolated intraepithelial lymphocytes of rhesus macaques after 3 h treatment, n = 4, **p < 0.01. **Fig. S4.** Effects of BCG and BCG-Nanocage on the viability of CD14+ macrophages, CD3+ T cells, CD20+ B cells and CD56+ NK cells in PBMC from rhesus after 6 day treatment. All cells were gated in lymphocytes and macrophages population of PBMC. (A) Effects of BCG and BCG-Nanocage on the viability of CD14 + macrophages, n = 3, Mean ± S.D, *p < 0.05. (B) Effects of BCG and BCG-Nanocage on the viability of CD3+ T cells, n = 3, Mean ± S.D, ***p < 0.001. (C) Effects of BCG and BCG-Nanocage on the viability of CD20+ B cells, n = 3, Mean ± S.D, *p < 0.05. (D) Effects of BCG and BCG-Nanocage on the viability of CD56 + NK cells, n = 3, Mean ± S.D, *p < 0.05. **Fig. S5.** Ex vivo activation and expansion of CD4+ CD161+ T cells in PBMC from rhesus macaques induced by co-stimulation with BCG-Nanocage and IL2. (A) Typical flow cytometry panel for CD4+ CD161+ cells gated in T cells from PBMC with or without indicated (co-)stimulations of BCG/IL2, BCG-Nanocage/IL2 or IL2 for 6 days. (B) Effects of indicated (co-)stimulations of BCG/IL2, BCG-Nanocage/IL2 or IL2 on CD4+ CD161+ cells in T cells after 6 day treatment, n = 3, *p < 0.05. (C) Typical flow cytometry panel for Granzyme B+ Perforin + cells gated in CD4+ CD161+ T cells from PBMC with or without indicated (co-)stimulations of BCG/IL2, BCG-Nanocage/IL2 or IL2 for 6 days. (D) Effects indicated (co-)stimulations of BCG/IL2, BCG-Nanocage/IL2 or IL2 on CD4+ CD161+ Granzyme B+ Perforin+ cells in T cells after 6 day treatment, n = 3, *p < 0.05. (E) Typical flow cytometry panel for Granulysin + IFN-γ + cells gated in CD4+ CD161+ T cells from PBMC with or without indicated (co-)stimulations of BCG/IL2, BCG-Nanocage/IL2 or IL2 for 6 days. (F) Effects of indicated (co-)stimulations of BCG/IL2, BCG-Nanocage/IL2 co- or IL2 on CD4+ CD161+ Granulysin + IFN-γ+ cells in T cells after 6 day treatment, n = 3, *p < 0.05. **Fig. S6.** Ex vivo activation and expansion of CD8+ cytotoxic T cells in PBMC from rhesus macaques induced by BCG-Nanocage and IL2 co-stimulation. (A) Typical flow cytometry panel for CD8 + NKG2C + cells gated in T cells from PBMC with or without (co-)stimulations using BCG/IL2, BCG-Nanocage/IL2 or IL2 for 6 days. (B) Typical flow cytometry panel for Granzyme B + Perforin + cells gated in CD8+ NKG2C+ T cells from PBMC with or without (co-)stimulations using BCG/IL2, BCG-Nanocage/IL2 or IL2 for 6 days. (C) Typical flow cytometry panel for Granulysin + IFN-γ + cells gated in CD8 + NKG2C + Granzyme B + Perforin + T cells from PBMC with or without (co-)stimulations using BCG/IL2, BCG-Nanocage/IL2 or IL2 for 6 days. (D) Effects of (co-)stimulations using BCG/IL2, BCG-Nanocage/IL2 or IL2 on CD8 + NKG2C + Granzyme B + Perforin + Granulysin + IFN-γ + cells in T cells after 6 day treatment, n = 3, **p < 0.01. **Fig. S7.** BCG and BCG-Nanocage immunization in macaques promote IFN-γ production in CD4+ and CD8+ T cells against stimulation co-stimulations with anti-CD28+ anti-CD49d and BCG-Nanocage. Typical flow images for intracellular IFN-γ levels in CD4+ T cells from PBMC of rhesus macaques before and after BCG immunization or BCG-Nanocage immunization with (A) anti-CD28/anti-CD49d stimulation or (B) anti-CD28/anti-CD49d and BCG-Nanocage stimulation. (C) Statistical results for anti-CD28/anti-CD49d and BCG-Nanocage stimulation induced intracellular IFN-γ levels in CD4+ T cells from PBMC of rhesus macaques before and after BCG immunization or BCG-Nanocage immunization, data were expressed as percentages of control, n = 4. Typical flow images for intracellular IFN-γ level in CD8+ T cells from PBMC of rhesus macaques before and after BCG immunization or BCG-Nanocage immunization with (D) anti-CD28/anti-CD49d stimulation or (E) anti-CD28/anti-CD49d and BCG-Nanocage co-stimulation. (F) Statistical results for anti-CD28/anti-CD49d and BCG-Nanocage stimulation induced changes of intracellular IFN-γ levels in CD8+ T cells from PBMC of rhesus macaques before and after BCG immunization or BCG-Nanocage immunization, data were expressed as percentages of control, n = 4. **Fig. S8.** BCG-Nanocage immunization in macaques promote IFN-γ production in CD4+ and CD8+ T cells against co-stimulation with anti-CD28+ anti-CD49d and BCG-Nanocage. Typical flow images for intracellular Granzyme B levels in CD4+ T cells from PBMC of rhesus macaques before and after BCG immunization or BCG-Nanocage immunization with (A) anti-CD28/anti-CD49d stimulation or (B) co-stimulation with anti-CD28/anti-CD49d and BCG-Nanocage. (C) Statistical results for co-stimulation anti-CD28/anti-CD49d and BCG-Nanocage induced changes of intracellular Granzyme B levels in CD4+ T cells from PBMC of rhesus macaques before and after BCG immunization or BCG-Nanocage immunization, data were expressed as percentages of control, n = 4. Typical flow images for intracellular Granzyme B levels in CD8+ T cells from PBMC of rhesus macaques before and after BCG immunization or BCG-Nanocage immunization with (D) anti-CD28/anti-CD49d stimulation or (E) co-stimulation with anti-CD28/anti-CD49d and BCG-Nanocage. (F) Statistical results for anti-CD28/anti-CD49d and BCG-Nanocage stimulation induced changes of intracellular Granzyme B levels in CD8+ T cells from PBMC of rhesus macaques before and after BCG immunization or BCG-Nanocage immunization, data were expressed as percentages of control, n = 4. **Fig. S9.** BCG-Nanocage boost immunization induce rapid peripheral Th1/Th1-like responses to Mtb challenge. Typical flow images for IFN-γ + CD4 + Th1 effector cells in the T cells of PBMC from (A) saline, (B) BCG-immunized and (C) BCG-Nanocage-immunized Rhesus before and after Mtb infection. Typical flow images for IFN-γ + CD8+ Th1-like effector cells in T cells in PBMC from (D) saline, (E) BCG-immunized and (F) BCG-Nanocage-immunized Rhesus before and after Mtb infection. (G) Changes of IFN-γ + CD4 + Th1 effector cells in the T cells of PBMC from saline, BCG-immunized and BCG-Nanocage-immunized Rhesus before and after Mtb infection. (H) Changes of IFN-γ + CD8 + Th1-like effector cells in T cells in PBMC from saline, BCG-immunized and BCG-Nanocage- immunized Rhesus before and after Mtb infection. **Fig. S10.** The swelling of hilar lymph nodes (HLN) in saline-treated but not in BCG- immunized or BCG-Nanocage-immunized rhesus macaques after Mtb challenge. Blue arrow indicated the swelling structures of HLN.

## Data Availability

The datasets used and/or analyzed in the current study are available from the corresponding author on reasonable request.
