# Supplementary material for: Nanocages engineered from Bacillus Calmette-Guerin facilitate protective Vγ2Vδ2 T cell immunity against Mycobacterium tuberculosis infection
Source: J Nanobiotechnology. 2022 Jan 15;20:36. doi: 10.1186/s12951-021-01234-3 (PMC8760571; doi:10.1186/s12951-021-01234-3)
Supplement: Supplementary file 1 — Additional file 1: Fig. S1. Size of BCG-Nanocage upon -80℃storage. Size distribution of BCG-Nanocage before and after 70 days storage at −80℃, samples were thawed at room temperature for size measurements. Fig. S2. BCG preferentially target macrophages (MΦ), but not T cells, B cells or endothelium ex vivo. (A) Confocal imaging for cellular uptake of GFP-BCG by macrophages (MΦ) from the freshly isolated bronchoalveolar lavage (BAL) fluid of rhesus macaques after 1 h treatment of GFP-BCG, followed with anti-human CD11b-APC antibody and anti-human CD3-PE antibody staining, scale bar: 20 μm. (B) Flow cytometry analysis for cellular uptake of GFP-BCG by macrophages, T cells, B cells and endothelium from the freshly isolated intraepithelium (IEL) of rhesus macaques after 1 h treatment. (C) Cellular uptake of GFP-BCG by macrophages, T cells, B cells and endothelium from the freshly isolated intraepithelial lymphocytes of rhesus macaques after 1 h treatment, n = 4, ***p < 0.001. Fig. S3. Comparison of BCG and BCG-Nanocage uptake by macrophages (MΦ), T cells, B cells and endothelium ex vivo. Cellular uptake of GFP-BCG or GFP-BCG-Nanocage by macrophages, T cells, B cells and endothelium from the freshly isolated intraepithelial lymphocytes of rhesus macaques after 1 h treatment, n = 4. Cellular uptake of GFP-BCG or GFP-BCG-Nanocage by macrophages, T cells, B cells and endothelium from the freshly isolated intraepithelial lymphocytes of rhesus macaques after 3 h treatment, n = 4, **p < 0.01. Fig. S4. Effects of BCG and BCG-Nanocage on the viability of CD14+ macrophages, CD3+ T cells, CD20+ B cells and CD56+ NK cells in PBMC from rhesus after 6 day treatment. All cells were gated in lymphocytes and macrophages population of PBMC. (A) Effects of BCG and BCG-Nanocage on the viability of CD14 + macrophages, n = 3, Mean ± S.D, *p < 0.05. (B) Effects of BCG and BCG-Nanocage on the viability of CD3+ T cells, n = 3, Mean ± S.D, ***p < 0.001. (C) Effects of BCG and BCG-Nanocage on the [file 12951_2021_1234_MOESM1_ESM.docx]

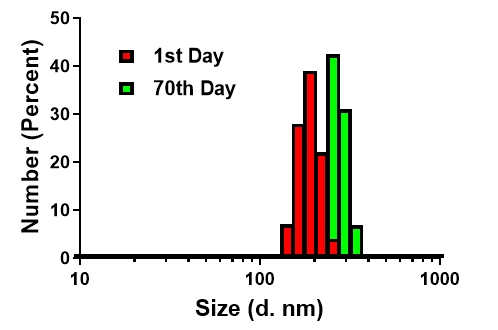


**Supplementary Materials. Fig.S1 Size of BCG-Nanocage upon** **-80℃storage.** Size distribution of BCG-Nanocage before and after 70 days storage at -80℃, samples were thawed at room temperature for size measurements.


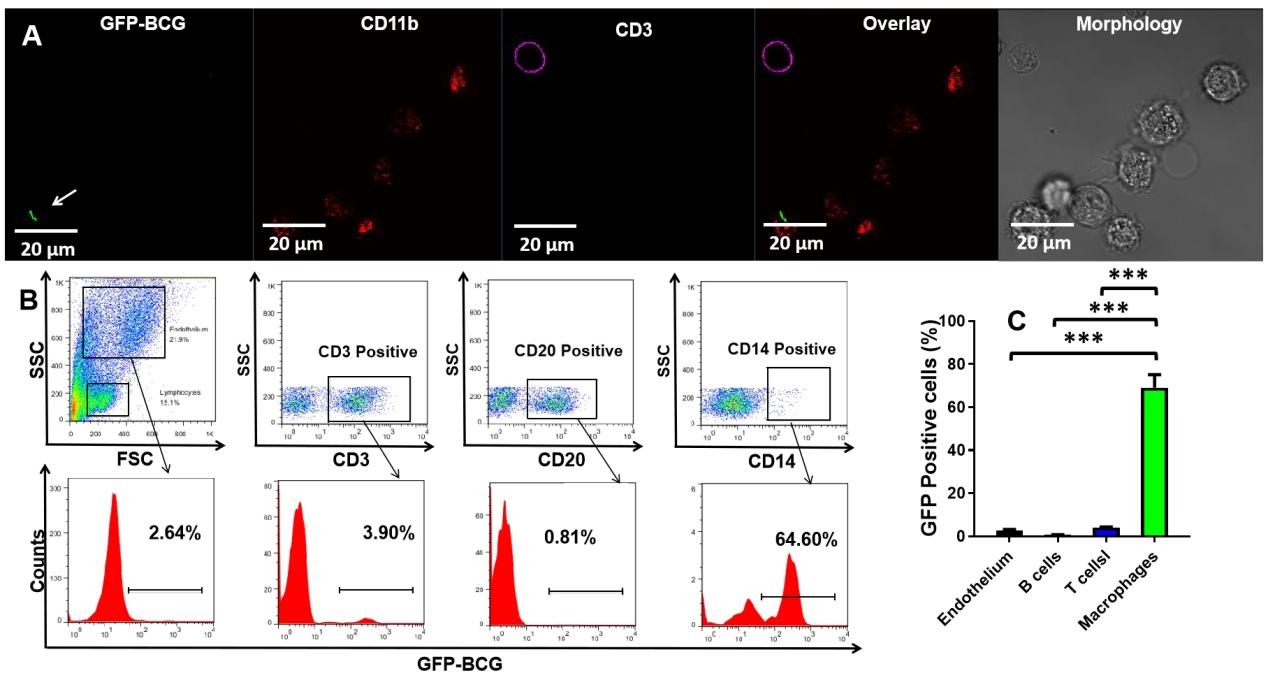


**Supplementary Materials. Fig.S2 BCG** **preferentially target macrophages (MΦ), but not T cells, B cells or endothelium *ex vivo*.**

(A) Confocal imaging for cellular uptake of GFP-BCG by macrophages (MΦ) from the freshly isolated bronchoalveolar lavage (BAL) fluid of rhesus macaques after 1 h treatment of GFP-BCG, followed with anti-human CD11b-APC antibody and anti-human CD3-PE antibody staining, scale bar: 20 μm.
(B) Flow cytometry analysis for cellular uptake of GFP-BCG by macrophages, T cells, B cells and endothelium from the freshly isolated intraepithelium (IEL) of rhesus macaques after 1 h treatment.
(C) Cellular uptake of GFP-BCG by macrophages, T cells, B cells and endothelium from the freshly isolated intraepithelial lymphocytes of rhesus macaques after 1 h treatment, n=4, ***p<0.001.


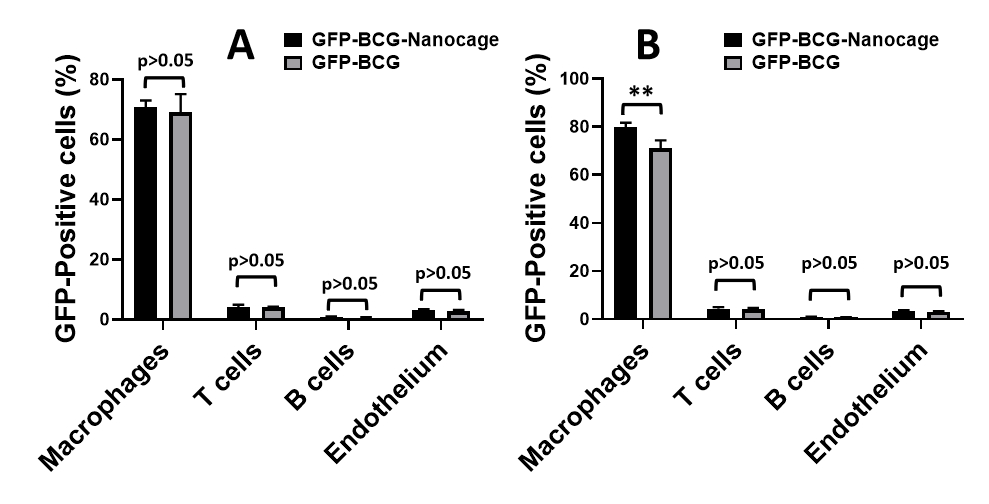


**Supplementary Materials. Fig.S3 Comparison of BCG and BCG-Nanocage uptake by macrophages (MΦ), T cells, B cells and endothelium *ex vivo***.

1. Cellular uptake of GFP-BCG or GFP-BCG-Nanocage by macrophages, T cells, B cells and endothelium from the freshly isolated intraepithelial lymphocytes of rhesus macaques after 1 h treatment, n=4.
2. Cellular uptake of GFP-BCG or GFP-BCG-Nanocage by macrophages, T cells, B cells and endothelium from the freshly isolated intraepithelial lymphocytes of rhesus macaques after 3 h treatment, n=4, **p<0.01.


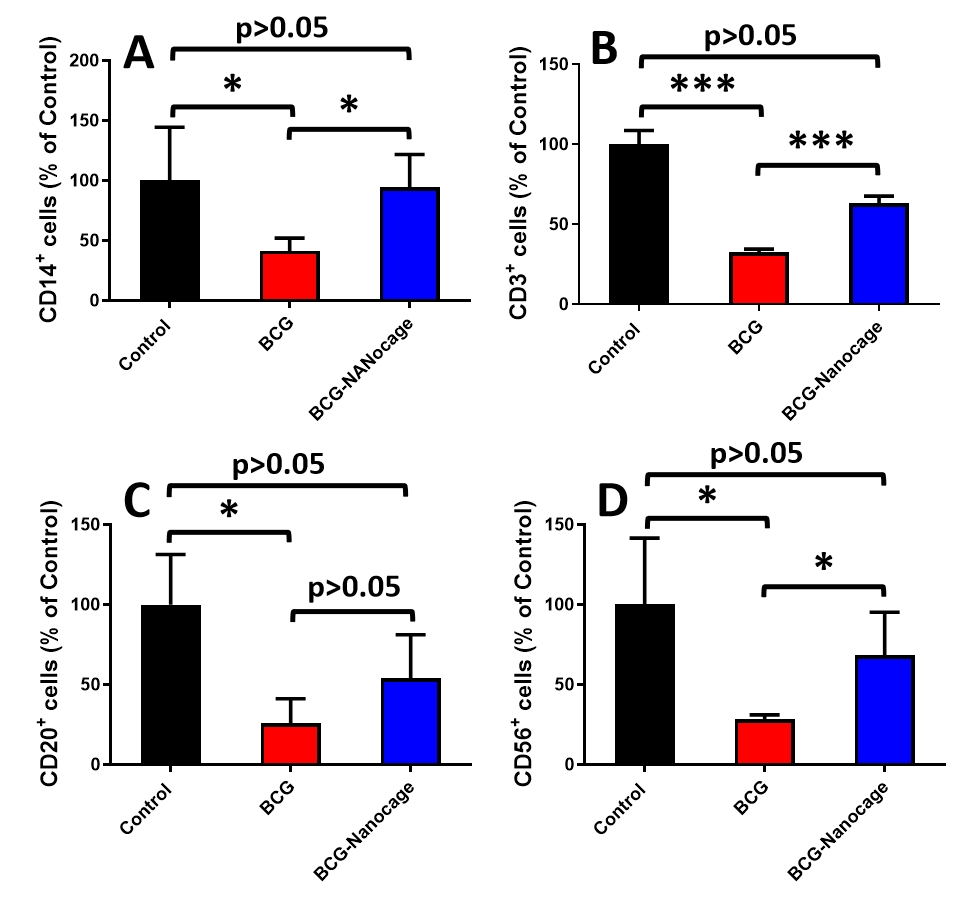
**Supplementary Materials. Fig.S4 Effects of BCG and BCG-Nanocage on the viability of CD14+ macrophages, CD3+ T cells, CD20+ B cells and CD56+ NK cells in PBMC from rhesus after 6 day treatment**. All cells were gated in lymphocytes and macrophages population of PBMC.

(A) Effects of BCG and BCG-Nanocage on the viability of CD14+ macrophages, n=3, Mean±S.D, *p<0.05.

(B) Effects of BCG and BCG-Nanocage on the viability of CD3+ T cells, n=3, Mean±S.D, ***p<0.001.

(C) Effects of BCG and BCG-Nanocage on the viability of CD20+ B cells, n=3, Mean±S.D, *p<0.05.

(D) Effects of BCG and BCG-Nanocage on the viability of CD56+ NK cells, n=3, Mean±S.D, *p<0.05.


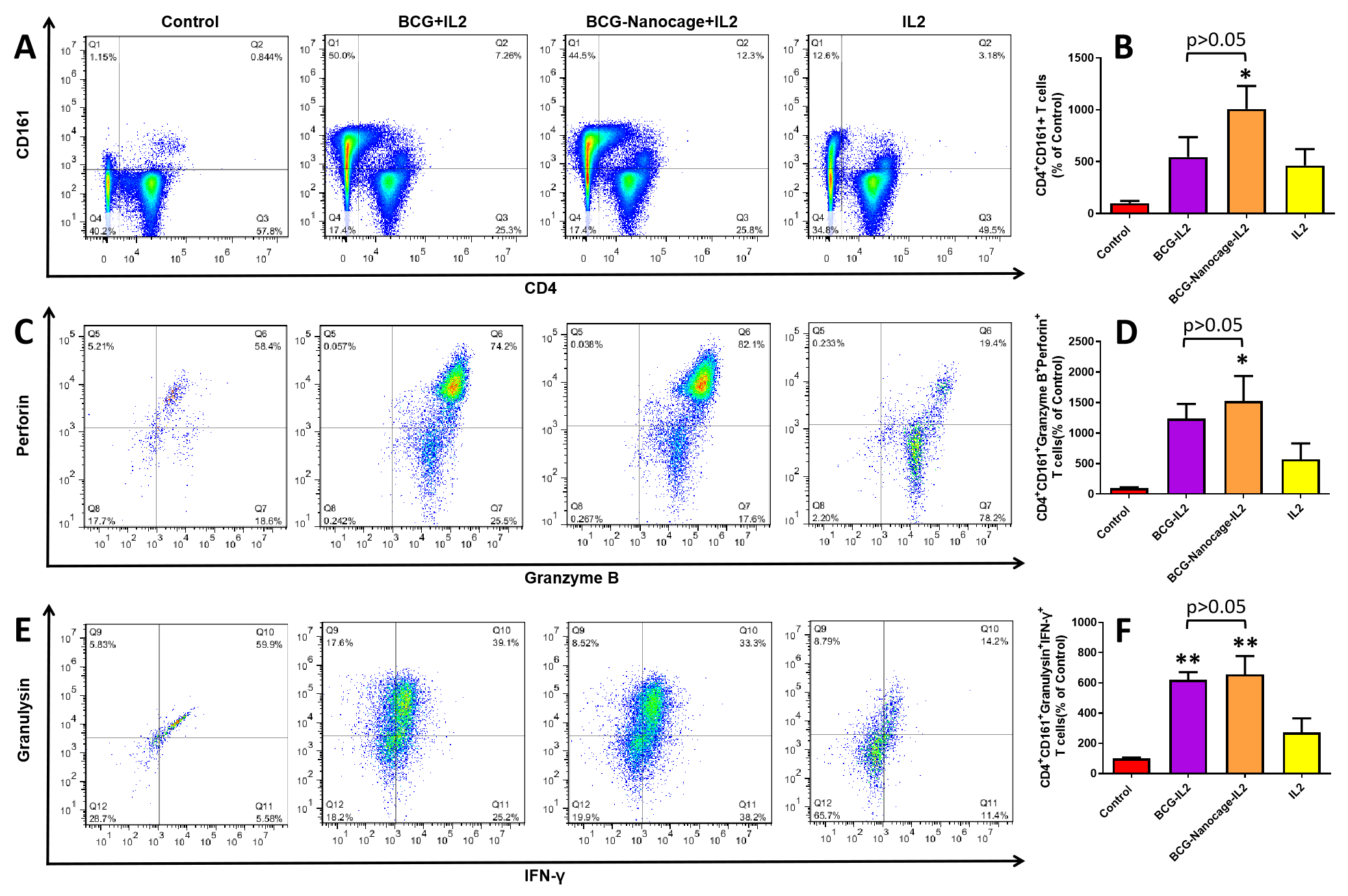
**Supplementary Materials. Fig.S5 *Ex vivo* activation and expansion of** **CD4+CD161+ T cells in PBMC from rhesus macaques induced by co-stimulation with BCG-Nanocage and IL2.**

(A) Typical flow cytometry panel for CD4+CD161+ cells gated in T cells from PBMC with or without indicated (co-)stimulations of BCG/IL2, BCG-Nanocage/IL2 or IL2 for 6 days.

(B) Effects of indicated (co-)stimulations of BCG/IL2, BCG-Nanocage/IL2 or IL2 on CD4+CD161+ cells in T cells after 6 day treatment, n=3, *p<0.05.

(C) Typical flow cytometry panel for Granzyme B+Perforin+ cells gated in CD4+CD161+ T cells from PBMC with or without indicated (co-)stimulations of BCG/IL2, BCG-Nanocage/IL2 or IL2 for 6 days.

(D) Effects indicated (co-)stimulations of BCG/IL2, BCG-Nanocage/IL2 or IL2 on CD4+CD161+Granzyme B+Perforin+ cells in T cells after 6 day treatment, n=3, *p<0.05.

(E) Typical flow cytometry panel for Granulysin+IFN-γ+ cells gated in CD4+CD161+ T cells from PBMC with or without indicated (co-)stimulations of BCG/IL2, BCG-Nanocage/IL2 or IL2 for 6 days.

(F) Effects of indicated (co-)stimulations of BCG/IL2, BCG-Nanocage/IL2 co- or IL2 on CD4+CD161+Granulysin+IFN-γ+ cells in T cells after 6 day treatment, n=3, *p<0.05.


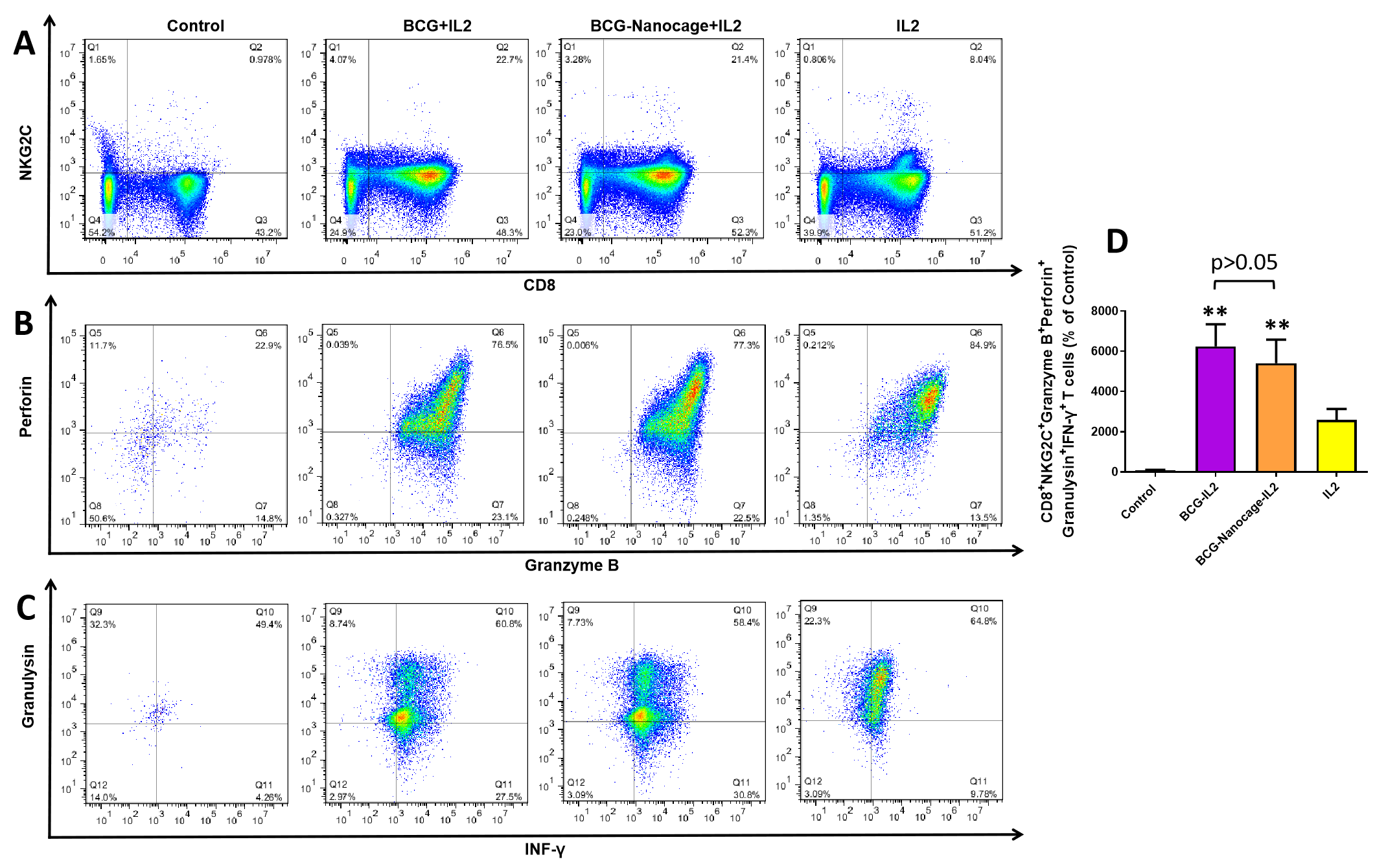
**Supplementary Materials. Fig.S6 *Ex vivo* activation and expansion of CD8+ cytotoxic T cells in PBMC from rhesus macaques induced by BCG-Nanocage and IL2 co-stimulation.**

(A) Typical flow cytometry panel for CD8+NKG2C+ cells gated in T cells from PBMC with or without (co-)stimulations using BCG/IL2, BCG-Nanocage/IL2 or IL2 for 6 days.

(B) Typical flow cytometry panel for Granzyme B+Perforin+ cells gated in CD8+NKG2C+ T cells from PBMC with or without (co-)stimulations using BCG/IL2 , BCG-Nanocage/IL2 or IL2 for 6 days.

(C) Typical flow cytometry panel for Granulysin+IFN-γ+ cells gated in CD8+NKG2C+Granzyme B+Perforin+ T cells from PBMC with or without (co-)stimulations using BCG/IL2, BCG-Nanocage/IL2 or IL2 for 6 days.

(D) Effects of (co-)stimulations using BCG/IL2, BCG-Nanocage/IL2 or IL2 on CD8+NKG2C+Granzyme B+Perforin+Granulysin+IFN-γ+ cells in T cells after 6 day treatment, n=3, **p<0.01.


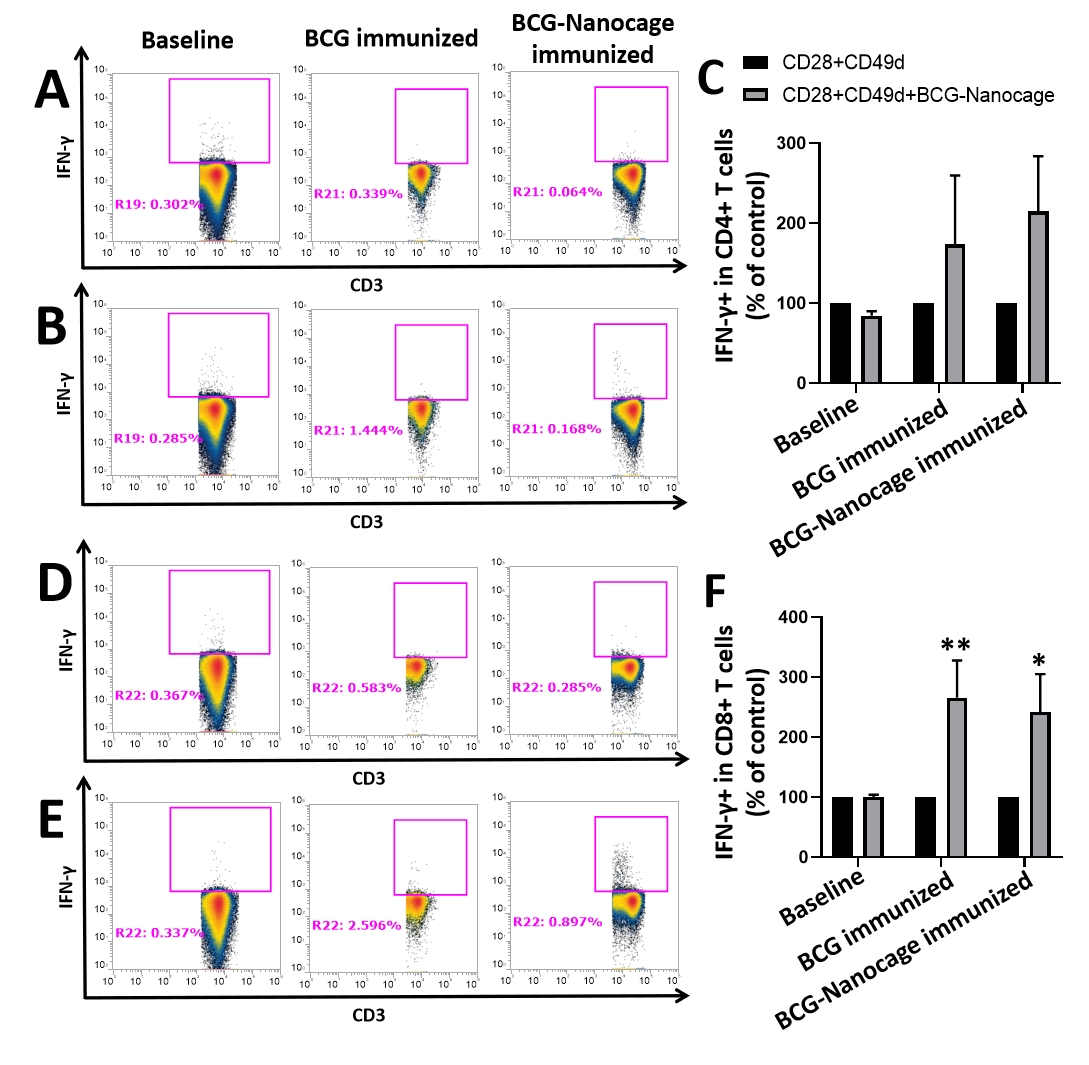


**Supplementary Materials. Fig.S7 BCG and BCG-Nanocage immunization in macaques promote IFN-γ production in CD4+ and CD8+ T cells against stimulation co-stimulations with anti-CD28+anti-CD49d and BCG-Nanocage.**

Typical flow images for intracellular IFN-γ levels in CD4+ T cells from PBMC of rhesus macaques before and after BCG immunization or BCG-Nanocage immunization with (A) anti-CD28/anti-CD49d stimulation or (B) anti-CD28/anti-CD49d and BCG-Nanocage stimulation.

(C) Statistical results for anti-CD28/anti-CD49d and BCG-Nanocage stimulation induced intracellular IFN-γ levels in CD4+ T cells from PBMC of rhesus macaques before and after BCG immunization or BCG-Nanocage immunization, data were expressed as percentages of control, n=4.

Typical flow images for intracellular IFN-γ level in CD8+ T cells from PBMC of rhesus macaques before and after BCG immunization or BCG-Nanocage immunization with (D) anti-CD28/anti-CD49d stimulation or (E) anti-CD28/anti-CD49d and BCG-Nanocage co-stimulation.

(F) Statistical results for anti-CD28/anti-CD49d and BCG-Nanocage stimulation induced changes of intracellular IFN-γ levels in CD8+ T cells from PBMC of rhesus macaques before and after BCG immunization or BCG-Nanocage immunization, data were expressed as percentages of control, n=4.


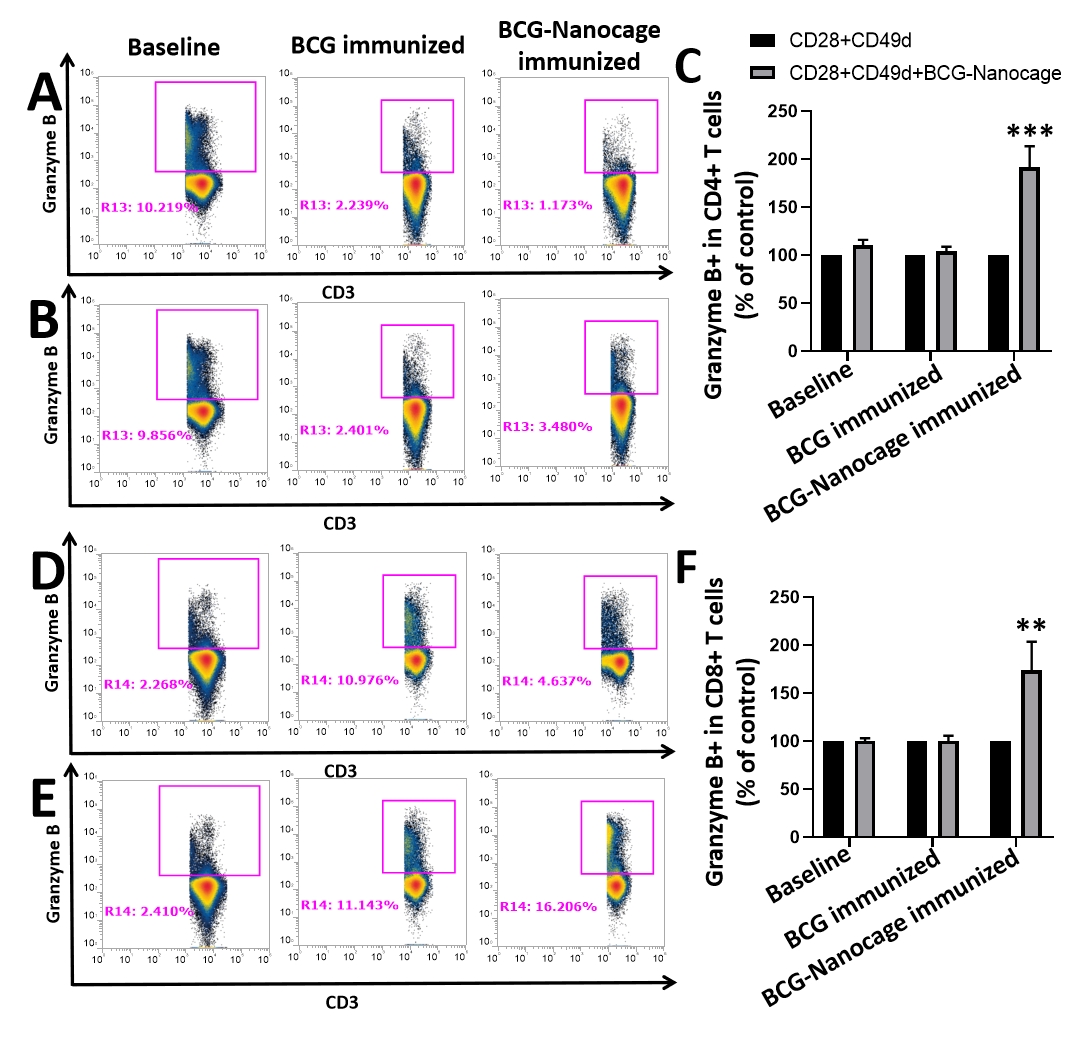


**Supplementary Materials. Fig.S8 BCG-Nanocage immunization in macaques promote IFN-γ production in CD4+ and CD8+ T cells against co-stimulation with anti-CD28+anti-CD49d and BCG-Nanocage.**

Typical flow images for intracellular Granzyme B levels in CD4+ T cells from PBMC of rhesus macaques before and after BCG immunization or BCG-Nanocage immunization with (A) anti-CD28/anti-CD49d stimulation or (B) co-stimulation with anti-CD28/anti-CD49d and BCG-Nanocage.

(C) Statistical results for co-stimulation anti-CD28/anti-CD49d and BCG-Nanocage induced changes of intracellular Granzyme B levels in CD4+ T cells from PBMC of rhesus macaques before and after BCG immunization or BCG-Nanocage immunization, data were expressed as percentages of control, n=4.

Typical flow images for intracellular Granzyme B levels in CD8+ T cells from PBMC of rhesus macaques before and after BCG immunization or BCG-Nanocage immunization with (D) anti-CD28/anti-CD49d stimulation or (E) co-stimulation with anti-CD28/anti-CD49d and BCG-Nanocage.

(F) Statistical results for anti-CD28/anti-CD49d and BCG-Nanocage stimulation induced changes of intracellular Granzyme B levels in CD8+ T cells from PBMC of rhesus macaques before and after BCG immunization or BCG-Nanocage immunization, data were expressed as percentages of control, n=4.


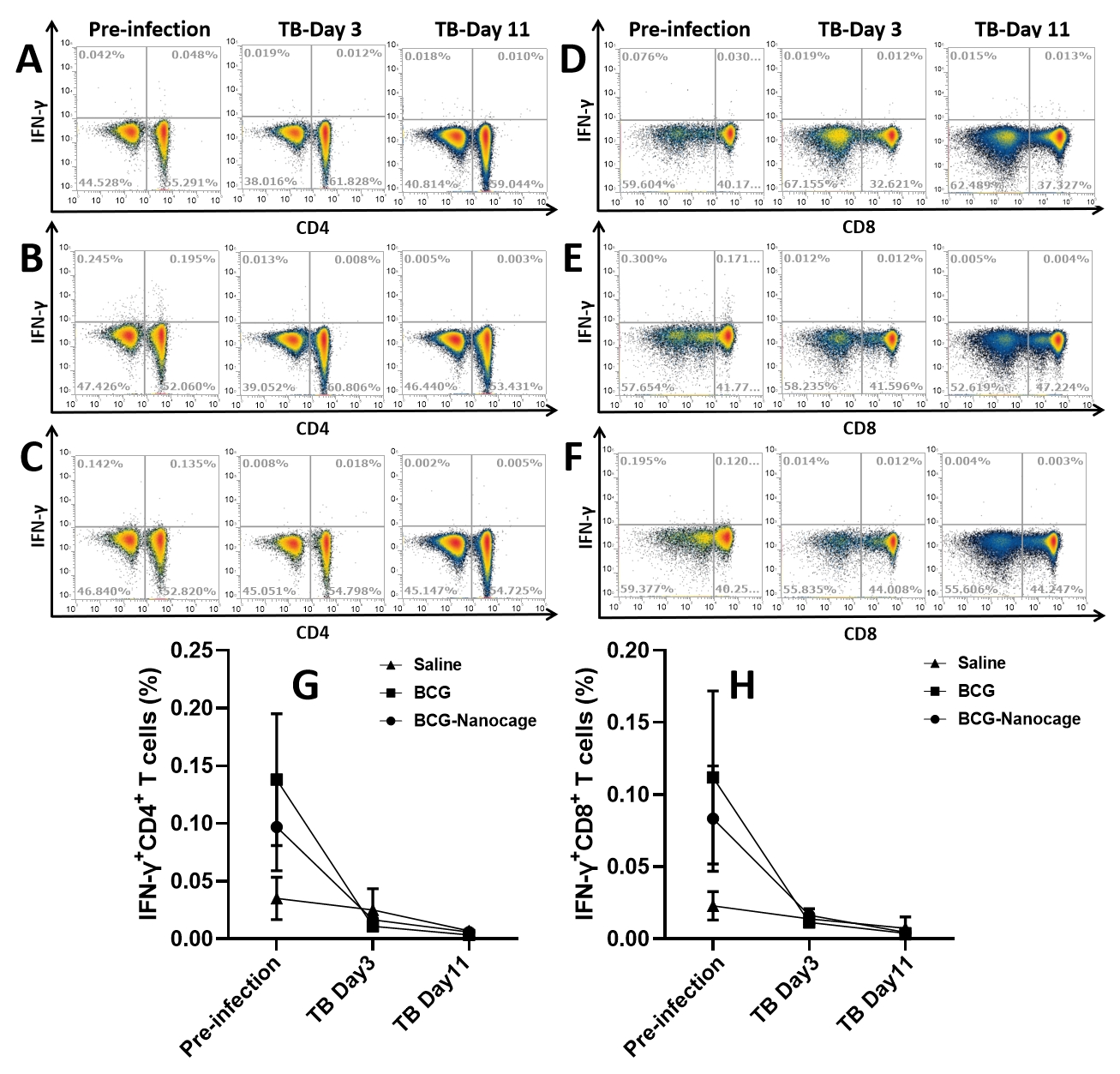


**Supplementary Materials. Fig.S9 BCG-Nanocage boost immunization induce rapid peripheral Th1/Th1-like responses to Mtb challenge.**

Typical flow images for IFN-γ+CD4+ Th1 effector cells in the T cells of PBMC from (A) saline, (B) BCG-immunized and (C) BCG-Nanocage-immunized Rhesus before and after Mtb infection.

Typical flow images for IFN-γ+CD8+ Th1-like effector cells in T cells in PBMC from (D) saline, (E) BCG-immunized and (F) BCG-Nanocage-immunized Rhesus before and after Mtb infection.

(G) Changes of IFN-γ+CD4+ Th1 effector cells in the T cells of PBMC from saline, BCG-immunized and BCG-Nanocage-immunized Rhesus before and after Mtb infection.

(H) Changes of IFN-γ+CD8+ Th1-like effector cells in T cells in PBMC from saline, BCG-immunized and BCG-Nanocage- immunized Rhesus before and after Mtb infection.


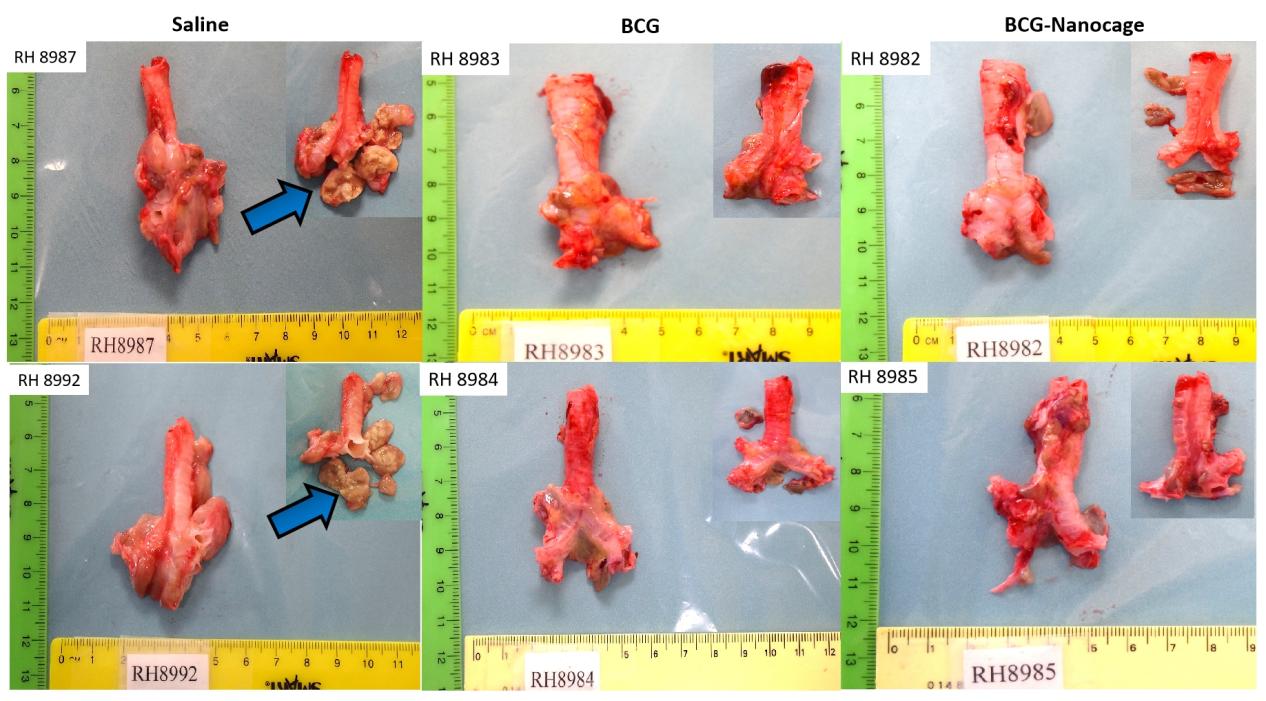


**Supplementary Materials. Fig.S10 The swelling of hilar lymph nodes (HLN) in saline-treated but not in BCG- immunized or BCG-Nanocage-immunized rhesus macaques after Mtb challenge.** Blue arrow indicated the swelling structures of HLN.
